# Pregnancy establishment in cattle without embryonic interferon tau

**DOI:** 10.1038/s41467-026-78446-4

**Published:** 2026-10-08

**Authors:** Asghar Ali, Valeri Zakhartchenko, Tuna Güngör, Stefan Nüske, Jan B. Stöckl, Sarah Hecken, Andreas Hauser, Stefan Krebs, Sven Reese, Marcel Suchowski, Robert Friedl, Sebastian Schwanitz, Armin M. Scholz, Andreas Blutke, Martina Protschka, Mathias Büttner, Thomas Fröhlich, Horst-Dieter Reichenbach, Eckhard Wolf

**Affiliations:** 1 Chair for Molecular Animal Breeding and Biotechnology, Gene Center and Department of Veterinary Sciences, LMU Munich, Munich, Germany; 2 Center for Innovative Medical Models (CiMM), LMU Munich, Oberschleißheim, Germany; 3 Livestock Center, Faculty of Veterinary Medicine, LMU Munich, Oberschleißheim, Germany; 4 Laboratory for Functional Genome Analysis (LAFUGA), Gene Center, LMU Munich, Munich, Germany; 5 Department of Veterinary Sciences, Chair of Anatomy, Histology and Embryology, LMU Munich, Munich, Germany; 6 Bavarian Health and Food Safety Authority (LGL), Oberschleißheim, Germany; 7 Vion Waldkraiburg GmbH, Waldkraiburg, Germany; 8 Institute of Veterinary Pathology, Center for Clinical Veterinary Medicine, LMU Munich, Munich, Germany; 9 Institute of Immunology, Faculty of Veterinary Medicine, University of Leipzig, Leipzig, Germany; 10 Institute for Animal Breeding, Bavarian State Research Center for Agriculture (LfL), Grub, Germany

**Keywords:** Embryology, Endocrine reproductive disorders, Genetic engineering

## Abstract

In ruminants, embryonic interferon tau (IFNT) is widely considered the indispensable signal for maternal recognition of pregnancy, driving endometrial gene expression to prevent luteolysis. However, this canonical paradigm has never been directly tested using genomic loss-of-function models. Here we show that IFNT is completely dispensable for pregnancy recognition, luteal maintenance, and full-term gestation in cattle. Using CRISPR-Cas9, we generate bovine fibroblasts lacking all functional copies of the multigene *IFNT* locus for somatic cell nuclear transfer. *IFNT*-null embryos develop into blastocysts in vitro at normal rates and exhibit typical elongation at day 18 post-transfer without inducing endometrial interferon-stimulated genes. Crucially, these *IFNT*-null conceptuses successfully prevent luteal regression, establish normal placentation, and progress through full-term gestations, culminating in the birth of healthy female calves. These findings provide the first direct genetic evidence that embryonic IFNT is dispensable for pregnancy recognition and establishment in cattle, fundamentally revising the long-standing model of ruminant reproduction and uncovering alternative embryo-maternal signaling mechanisms.

## Abstract (web summary)

A study in cattle demonstrates that early pregnancy establishment, fetal development, and the birth of live calves occur without embryonic interferon tau, overturning a fundamental paradigm of ruminant reproductive biology.

## Introduction

Early pregnancy loss remains a major limitation to reproductive efficiency in ruminants, with the vast majority of embryonic mortalities occurring within the first 3 weeks following conception^1^. During this critical peri-implantation phase, the conceptus, comprising the embryo proper and its extraembryonic membranes, undergoes rapid growth and elongation, and synchronously initiates reciprocal molecular communication with the maternal endometrium^2,3^. This intricate embryo-maternal dialogue culminates in maternal recognition of pregnancy (MRP)^4,5^, the critical physiological process by which the conceptus signals its presence to forestall luteolysis and ensure continued maternal support. In ruminants, this primary survival signal has long been attributed solely to interferon tau (IFNT), a type I interferon secreted exclusively by the mononuclear trophectoderm cells of the elongating conceptus^2,6^, and universally regarded as the indispensable mediator of MRP^7–10^.

IFNT was first identified in 1979 in cultured sheep conceptuses and originally termed ovine trophoblast protein-1 (oTP-1) or trophoblastin^11–15^. Its identity as a type I interferon was subsequently established by expression cloning of its cDNA, first in sheep^16^ and later in cattle^17^. In cattle, IFNT is secreted from approximately days 7 to 28 of gestation, with peak production occurring around days 18–20^18^. Acting through the classical type I interferon receptor complex (IFNAR1/IFNAR2), IFNT was reported to exert simultaneous autocrine actions on the conceptus, paracrine actions on the endometrium, and endocrine actions on the corpus luteum (reviewed in refs. ^7,10^). Its antiluteolytic effect is thought to be mediated by the interferon regulatory factor 2 (IRF2)-dependent inhibition of estrogen receptor alpha (*ESR1*) and oxytocin receptor (*OXTR*) upregulation within the endometrial luminal epithelium^19,20^. According to the current model, this effectively suppresses oxytocin-induced prostaglandin F₂α (PGF₂α) pulses, thereby averting luteolysis. Concurrently, IFNT is thought to target the corpus luteum directly to promote luteal cell survival, stabilize vascular integrity, and attenuate luteolytic signaling, thereby complementing its uterine actions to extend luteal lifespan during early pregnancy^21^. Beyond its antiluteolytic action, IFNT orchestrates a broad transcriptional response in the endometrium, including the robust activation of interferon-stimulated genes (ISGs) such as *ISG15, MX1, MX2, OAS1*, and *IRF9*, alongside the cell-type-specific modulation of pathways implicated in conceptus elongation, implantation, and immune regulation^2,5,7,10,22,23^. In cattle, endometrial ISG expression reaches peak levels around days 16–18 of gestation, corresponding to maximal embryonic IFNT signaling during the critical MRP window^10,24,25^.

Cattle possess multiple paralogous copies of the *IFNT* gene, with at least three validated coding sequences mapped to chromosome 8: *IFN-tau-c1* (also termed *IF1BE2*), *IFNT2* (also known as *TP-1*, *IFNT*, or *IFN-tau-c2*), and *IFNT3*, all of which encode the 195-amino acid IFNT protein^26–28^. Despite decades of intensive study and the universal acceptance of IFNT as the canonical MRP signal in ruminants, definitive loss-of-function evidence demonstrating its absolute requirement has remained elusive. Although the generation and fertility status of sheep lacking the type I interferon receptor subunit 2 (IFNAR2) has been previously reported^29,30^, the complete genetic ablation of IFNT, and the resulting disruption of potential IFNT-receptor interaction at the maternal-embryonic interface, had not been achieved in any ruminant species prior to this study.

Here, we directly tested the long-standing paradigm of IFNT being an obligatory embryonic signal in MRP by generating bovine embryos with complete ablation of all functional *IFNT* gene copies. Using somatic cell nuclear transfer (SCNT) with *IFNT*-knockout donor cells, we evaluated the developmental competence of *IFNT*-null embryos to establish and maintain pregnancy following transfer to synchronized recipients.

## Results

### Generation of bovine embryos with targeted deletions of *IFNT* genes

**Fig. 1:**
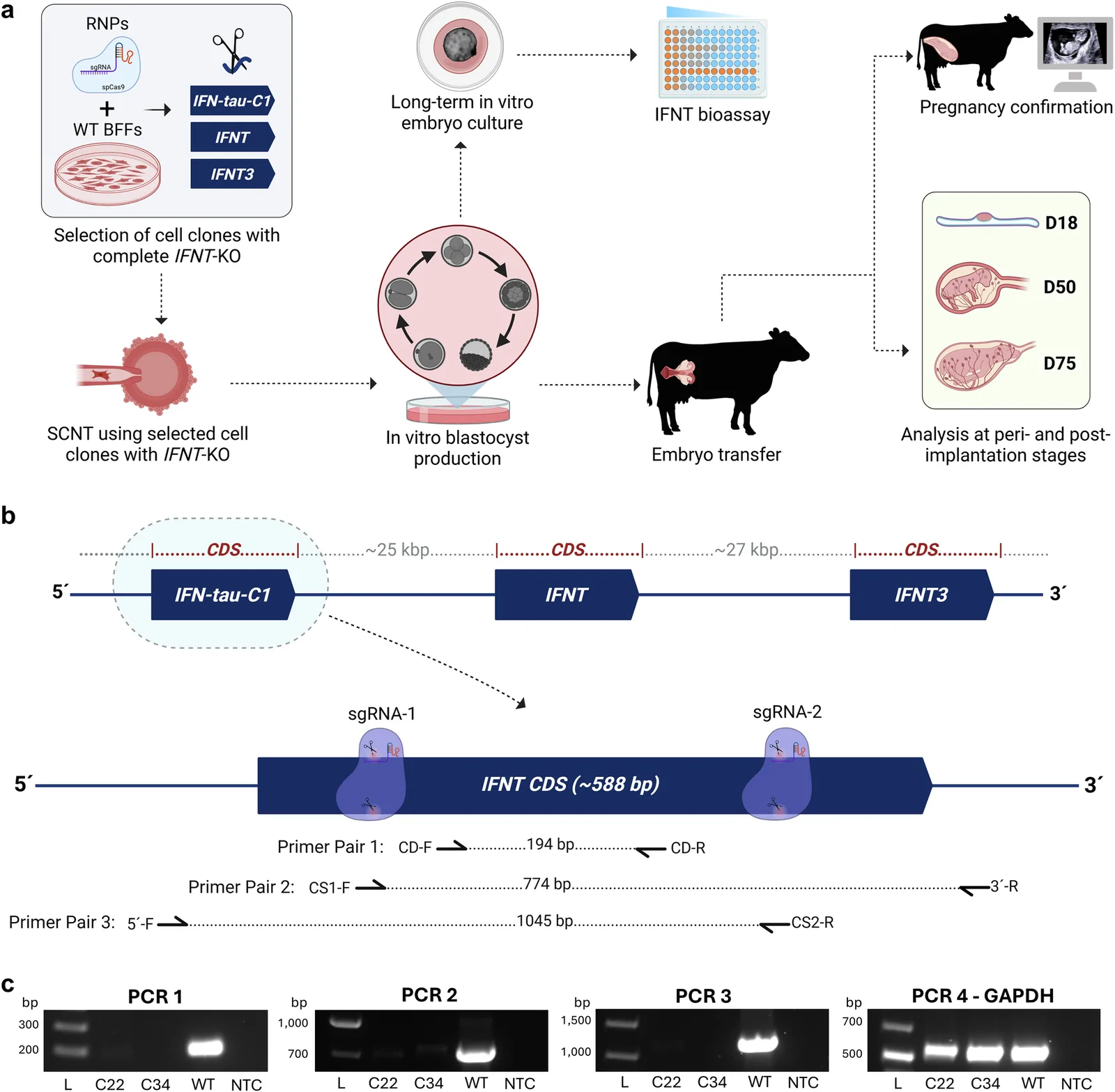
Study design and generation of *IFNT*-knockout donor cells. **a** Schematic overview of the experimental strategy. Bovine fetal fibroblasts (BFFs) were genome-edited to delete all *IFNT* alleles by utilizing a CRISPR-Cas9 system. Two *IFNT*-specific sgRNAs complexed with Cas9 recombinant protein as RNP complexes were delivered into BFFs via electroporation, followed by single-cell expansion. The resulting single-cell clones were first validated in vitro to confirm complete genetic deletion of *IFNT* before being utilized as nuclear donors for SCNT. Reconstructed embryos from the selected clones were evaluated for IFNT secretion during extended in vitro culture, and the blastocysts from verified clones were transferred to synchronized recipients to analyze pregnancy establishment at defined gestational stages. **b**, Organization of the bovine *IFNT* multigene locus showing the locations of the two sgRNA cut sites and the three PCR assays used for genotyping. Primer Pair 1 (PCR 1) targets the *IFNT* coding sequence (CDS) between the two cut sites; Primer Pair 2 (PCR 2) spans the sgRNA1 cut site to the 3′ untranslated region (UTR); Primer Pair 3 (PCR 3) spans the 5′-UTR to the sgRNA2 cut site. **c**, PCR-based genotyping of candidate single-cell clones (C22 and C34) using genomic DNA. The absence of *IFNT*-specific amplicons across all three assays, while retaining the *GAPDH* control band, confirms the complete deletion of all detectable *IFNT* copies. Images are representative of 2 independent runs with similar results. **a**, **b** Created in BioRender. Ali, A. (2026) https://BioRender.com/3g3taku. Source data for **c** are provided in the Source Data file. KO, knockout; CRISPR, clustered regularly interspaced short palindromic repeats; sgRNAs, single-guide RNAs; RNP, ribonucleoprotein complex; SCNT, somatic cell nuclear transfer; PCR, polymerase chain reaction; L, DNA ladder; WT, wild-type; NTC, no-template control.

To determine whether conceptus-derived IFNT is required for pregnancy establishment in cattle, we utilized a CRISPR-Cas9 genome-editing approach to target the multigene *IFNT* locus. Two *IFNT*-specific single-guide RNAs (sgRNAs) complexed with Cas9 recombinant protein as ribonucleoprotein (RNP) complexes were delivered into bovine fetal fibroblast cells (BFFs) via electroporation, followed by single-cell expansion. The genome-edited single-cell clones lacking all functional *IFNT* alleles were used as nuclear donors for SCNT. Following verification of the absolute absence of IFNT protein secretion during extended in vitro culture, SCNT-derived *IFNT*-knockout (KO) or wild-type (WT) embryos were transferred to estrus-synchronized recipients to systematically assess different stages of pregnancy (Fig. 1a).

Candidate *IFNT*-KO single-cell clones were screened by three PCR assays performed on genomic DNA, targeting distinct regions of all functional *IFNT* gene copies (Fig. 1b). PCR 1 amplified the core *IFNT* coding sequence between the two sgRNA cut sites, and loss of this amplicon indicated deletion of the shared coding sequence (CDS). PCR 2 spanned from a forward primer overlapping the sgRNA1 cut site to a reverse primer in the 3′-UTR, and the absence of this product confirmed disruption at the sgRNA1 target site. PCR 3 amplified from the 5′-UTR to a reverse primer overlapping the sgRNA2 cut site, and loss of this amplicon indicated disruption at the sgRNA2 site. *GAPDH* amplification was used as an internal control. Two independent cell clones (22 and 34) lacked all three *IFNT*-specific amplicons while retaining the *GAPDH* band, confirming complete deletion of all detectable *IFNT* copies (Fig. 1c). These validated *IFNT*-KO clones were used for all subsequent in vitro and in vivo experiments.

### Normal in vitro development of *IFNT*-null embryos

**Fig. 2:**
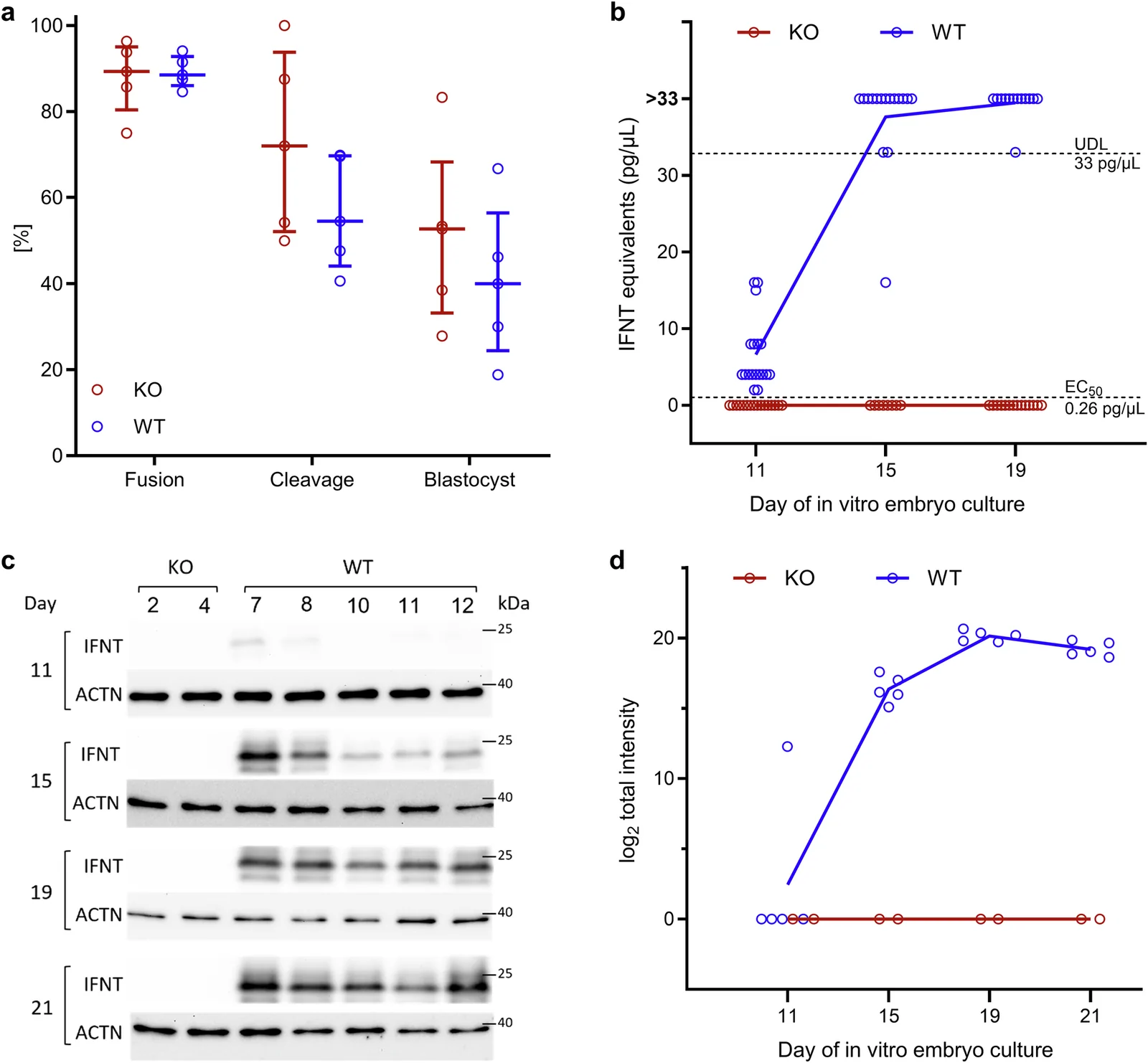
In vitro embryo culture and confirmation of *IFNT* ablation. **a** Developmental competence of *IFNT*-KO and WT embryos generated by SCNT. Center lines represent the median, and error bars represent the interquartile range (25th to 75th percentiles). Data points depict individual biological replicates (*n* = 5 independent SCNT sessions per group; total reconstructed embryos: *n* = 212 *IFNT*-KO, *n* = 144 WT; unit of study = individual SCNT session). Stage percentages for fusion rate (*p* > 0.99), cleavage rate (*p* = 0.22), and blastocyst formation rate (*p* = 0.55) were comparable between groups (two-sided Mann-Whitney *U* test). Detailed embryo numbers and developmental outcomes for each session are provided in Supplementary Table 1. **b** Cytopathic-effect (CPE) inhibition assay assessing IFNT antiviral activity in embryo-conditioned media collected from individually cultured embryos at indicated time points. Media from *IFNT*-KO embryos showed no detectable antiviral activity, whereas media from WT embryos exhibited robust IFNT activity that increased with embryo age. **c** Western blot analysis of IFNT protein in embryo-conditioned media from SCNT-derived *IFNT*-KO embryos (*n* = 2; samples 2 and 4) and *IFNT*-intact embryos (*n* = 5) generated by SCNT (sample 7) or IVF (samples 8, 10, 11, and 12). IFNT protein was undetectable in *IFNT*-KO samples but readily detected in WT samples. The samples were collected from independent biological replicates across separate blind experiments, demonstrating biological reproducibility without pooling. **d** Peptide-based quantitative LC-MS/MS analysis of IFNT abundance in the same conditioned media samples. IFNT peptides were absent from *IFNT*-KO media and abundant in WT media, with peak levels observed at day 19 of in vitro culture. **Note:** For Western blot and LC-MS/MS analyses (**c**, **d**), conditioned media were collected longitudinally from individually cultured embryos in an independent SCNT session. Only two SCNT-derived *IFNT*-KO embryos survived to day 21; data from these longest-surviving embryos are shown. The antiviral bioassay (**b**) included substantially more embryos and confirmed the complete absence of IFNT activity across all KO samples. Because embryos were cultured individually, all IFNT values in **b**–**d** represent secretion per embryo. Source data for **a**–**d** are provided in the Source Data file. KO, knockout; WT, wild-type; UDL, upper detection limit; EC₅₀, half-maximal effective concentration.

To assess whether *IFNT* ablation affects preimplantation development, *IFNT*-KO and WT embryos were generated by SCNT across five independent sessions (*n* = 5 biological replicates per group; Fig. 2a). In total, these experiments yielded 212 reconstructed *IFNT*-KO embryos and 144 WT embryos, with each data point in Fig. 2a representing the percentage of embryos progressing to a given stage within a single session. Fusion rates (median 89.3% KO vs. 88.5% WT; *p* > 0.99), cleavage rates (median 72.0% KO vs. 54.5% WT; *p* = 0.22), and blastocyst formation rates (median 52.7% KO vs. 40.0% WT; *p* = 0.55) were comparable between groups. These results indicate that *IFNT* knockout does not compromise in vitro preimplantation development. Detailed embryo numbers and developmental outcomes for each session are provided in Supplementary Table 1.

Microscopic evaluation of the blastocysts was performed daily; early blastocysts were first observed on day 5, expanded blastocysts on day 6, and hatching or fully hatched blastocysts on day 7–8 (Supplementary Fig. 1a), with no difference in developmental timing between the *IFNT*-KO and WT groups. High-quality hatched blastocysts were selected on day 7 for transfer to synchronized recipients. Some of the remaining blastocysts were utilized for long-term in vitro culture experiments, with each embryo cultured individually in an isolated microdrop. It is important to note that the long-term in vitro cultures were performed solely to assess IFNT secretion into the medium and were not intended to evaluate the effect of IFNT loss on post-blastocyst developmental potential. While a bovine embryo cannot develop normally under the currently employed in vitro conditions, the trophoblast cells proliferate and secrete IFNT^31^. Microscopic examination of embryos after extended culture confirmed the expected morphology (Supplementary Fig. 1b).

To evaluate IFNT bioactivity, SCNT-derived *IFNT*-KO embryos and *IFNT*-intact embryos derived by in vitro fertilization (IVF) were cultured in vitro until day 19, and embryo-conditioned media were collected at defined time points for viral cytopathic effect (CPE) inhibition assays. Conditioned media from *IFNT*-KO embryos showed no detectable antiviral activity at any stage, whereas media from IVF embryos exhibited robust IFNT-dependent antiviral activity that increased with time in culture (Fig. 2b). These results were independently confirmed in a long-term culture experiment in which SCNT-derived *IFNT*-KO embryos and WT embryos, generated either by SCNT from WT cells or by IVF, were maintained in vitro through day 21. Western blot analysis revealed a complete absence of IFNT protein in conditioned media from *IFNT*-KO embryos, while strong IFNT signals were detected in media from both SCNT- and IVF-derived WT embryos (Fig. 2c). Peptide-based quantification of IFNT through mass spectrometry of the same samples corroborated these findings, demonstrating a complete absence of IFNT peptides in *IFNT*-KO media and high IFNT abundance in WT media, with peak levels observed at day 19 of in vitro culture (Fig. 2d, and Supplementary Data 1a, b).

### Elongation of *IFNT*-KO embryos in the absence of an endometrial interferon response

**Fig. 3:**
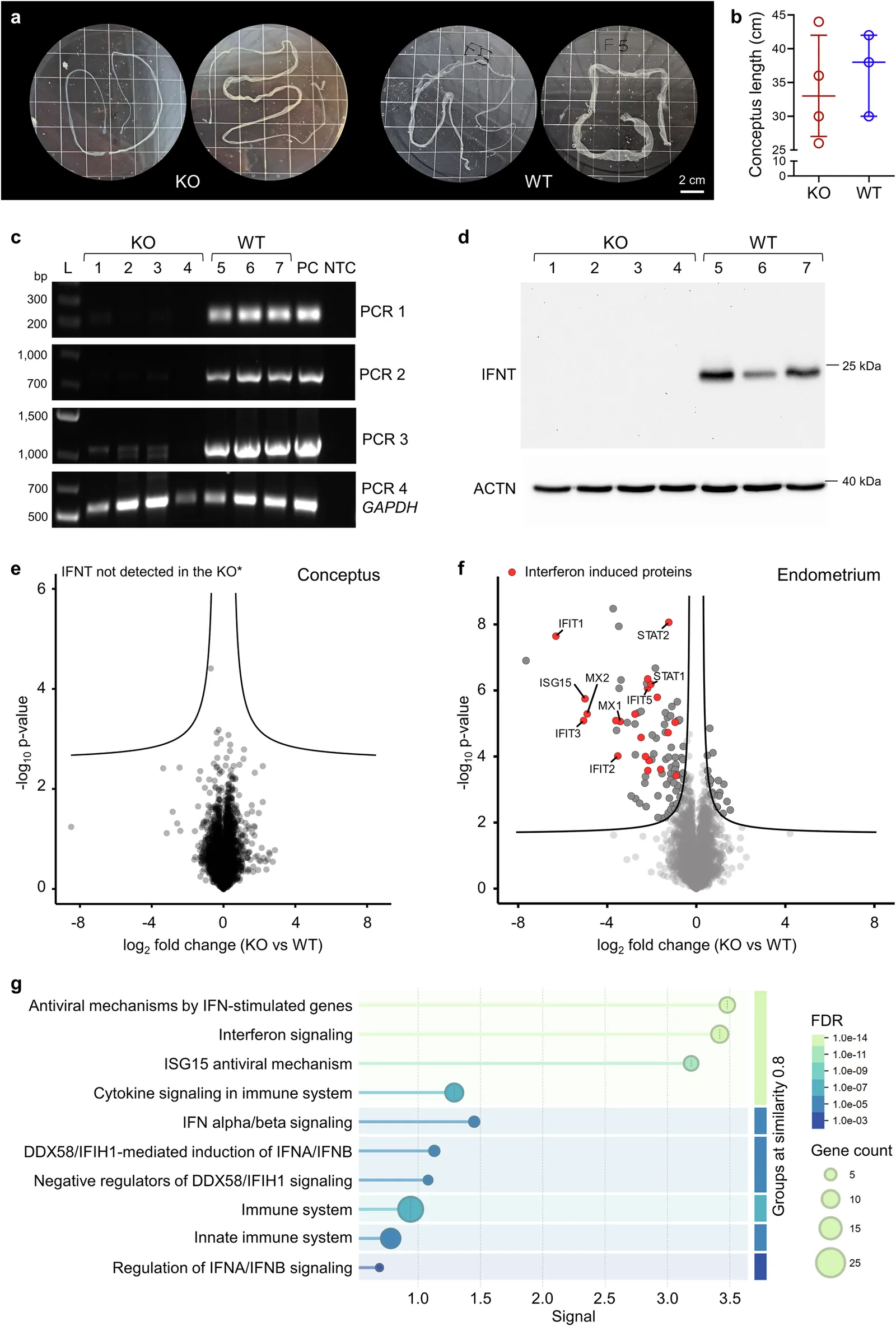
Early pregnancy establishment in the absence of conceptus-derived IFNT. **a** Representative images of day-18 conceptuses recovered from recipients carrying SCNT-derived *IFNT*-KO or WT embryos. **b** Length of day-18 *IFNT*-KO and WT conceptuses. The center line represents the median, and error bars represent the interquartile range (25th to 75th percentiles). Data points depict individual biological replicates (*n* = 4 *IFNT*-KO conceptuses and *n* = 3 WT conceptuses, recovered from independent recipient heifers). Groups were compared using a two-sided Mann–Whitney *U* test. No significant difference was observed between the groups (*p* = 0.71). **c** PCR-based genotyping of day-18 conceptuses with three primer sets targeting distinct regions of the *IFNT* locus. All *IFNT*-specific amplicons were absent in *IFNT*-KO conceptuses, while *GAPDH* amplification served as an internal control. **d** Western blot analysis of IFNT protein in day-18 conceptus tissues (*n* = 4 *IFNT*-KO and *n* = 3 WT). IFNT protein was detected in WT but was absent in *IFNT*-KO conceptuses. Samples assayed in 3c and 3d represent independent biological replicates derived from separate experiments. The western blot assay was independently repeated twice with similar results. **e** Quantitative proteomic analysis of day-18 conceptus tissues (*n* = 4 *IFNT*-KO and *n* = 3 WT). Global protein abundance profiles were comparable between *IFNT*-KO and WT cohorts; *IFNT*-derived peptides were detected exclusively in WT samples. **f** Proteomic profiling of endometrial tissue (*n* = 4 per group) from recipients carrying *IFNT*-KO or WT conceptuses. Interferon-stimulated proteins were markedly reduced in endometria associated with *IFNT*-KO conceptuses. Data in **e** and **f** were analyzed using a two-tailed Student’s *t*-test with a permutation-based false discovery rate (FDR) cutoff of 0.05 and *s*_0_ parameter of 0.1. Black hyperbolic curves indicate the significance threshold (FDR < 0.05). **g** Functional enrichment analysis of proteins downregulated in endometrium associated with *IFNT*-KO embryos, showing significant enrichment of interferon-related pathways, including type I interferon signaling and ISG15-mediated antiviral responses. Signal represents the weighted harmonic mean between the observed/expected ratio and -log (FDR); Gene count represents the number of proteins included in the enriched terms. Source data for **b**–**d** are provided in the Source Data file. KO, knockout; WT, wild-type; PC, positive control; L, DNA ladder; NTC, no-template control. *Details of all *IFNT*-specific peptides used and their results are provided in Supplementary Data 3.

Following robust in vitro validation of *IFNT* ablation, the requirement of conceptus-derived IFNT for early pregnancy establishment was assessed in vivo. *IFNT*-KO and WT SCNT-derived blastocysts were transferred into synchronized recipient heifers (2-3 embryos per recipient). Recipients were slaughtered at gestational day 18, and conceptuses were recovered by uterine flushing, followed by collection of endometrial biopsies (Fig. 3a). Conceptus recovery was 100% in the *IFNT*-KO group and 50% in the WT group, yielding a total of four *IFNT*-KO and three *IFNT*-intact conceptuses. Despite the complete absence of IFNT, no significant differences in conceptus elongation were observed between groups (*p* = 0.71), with mean conceptus lengths of 34.0 cm in the KO group and 36.6 cm in the WT group (Fig. 3b). PCR-based genotyping of recovered conceptuses confirmed the absence of all *IFNT* target regions in the KO group, verifying stable and complete *IFNT* deletion (Fig. 3c). Consistent with these findings, western blot analysis failed to detect IFNT protein in *IFNT*-KO conceptuses, whereas robust IFNT expression was observed in WT conceptuses (Fig. 3d).

Quantitative proteomic analysis of conceptus tissues revealed no global differences in protein abundance between *IFNT*-KO and WT groups (Fig. 3e, and Supplementary Data 2a, b); notably, no IFNT-derived peptides were detected in any of the *IFNT*-KO samples (Supplementary Data 3). Complementary transcriptomic profiling of the same conceptuses likewise showed no significant differences in gene expression between groups (Supplementary Data 4a, b). In addition, targeted analysis of prostaglandins in conceptus tissues revealed no differences in the levels of PGD₂, PGE₁, PGE₂, PGF₁α, or PGF₂α (Supplementary Fig. 2). Similarly, transcript and protein levels of the prostaglandin E synthases (PTGES, PTGES2, and PTGES3) were comparable between the two genotypes (Supplementary Data 5), indicating that the absence of IFNT does not trigger compensatory upregulation of prostaglandin synthesis or any other compensatory responses within the conceptus.

In contrast, proteomic profiling of the corresponding endometrium samples revealed marked differences between groups. Endometria associated with *IFNT*-KO conceptuses exhibited a pronounced reduction in interferon-stimulated proteins compared to those associated with WT conceptuses (Fig. 3f, and Supplementary Data 6a, b). Pathway enrichment analysis of downregulated proteins in the *IFNT*-KO endometrium identified significant enrichment for IFNT-associated pathways, including interferon signaling, ISG15-mediated antiviral mechanisms, IFNA/B signaling, and regulation of type I interferon responses (Fig. 3g, and Supplementary Data 7). These data demonstrate that, although *IFNT*-KO conceptuses fail to elicit the canonical uterine interferon response, they nevertheless elongate normally and establish early pregnancy.

### Establishment and progression of pregnancy in the absence of embryonic IFNT

**Fig. 4:**
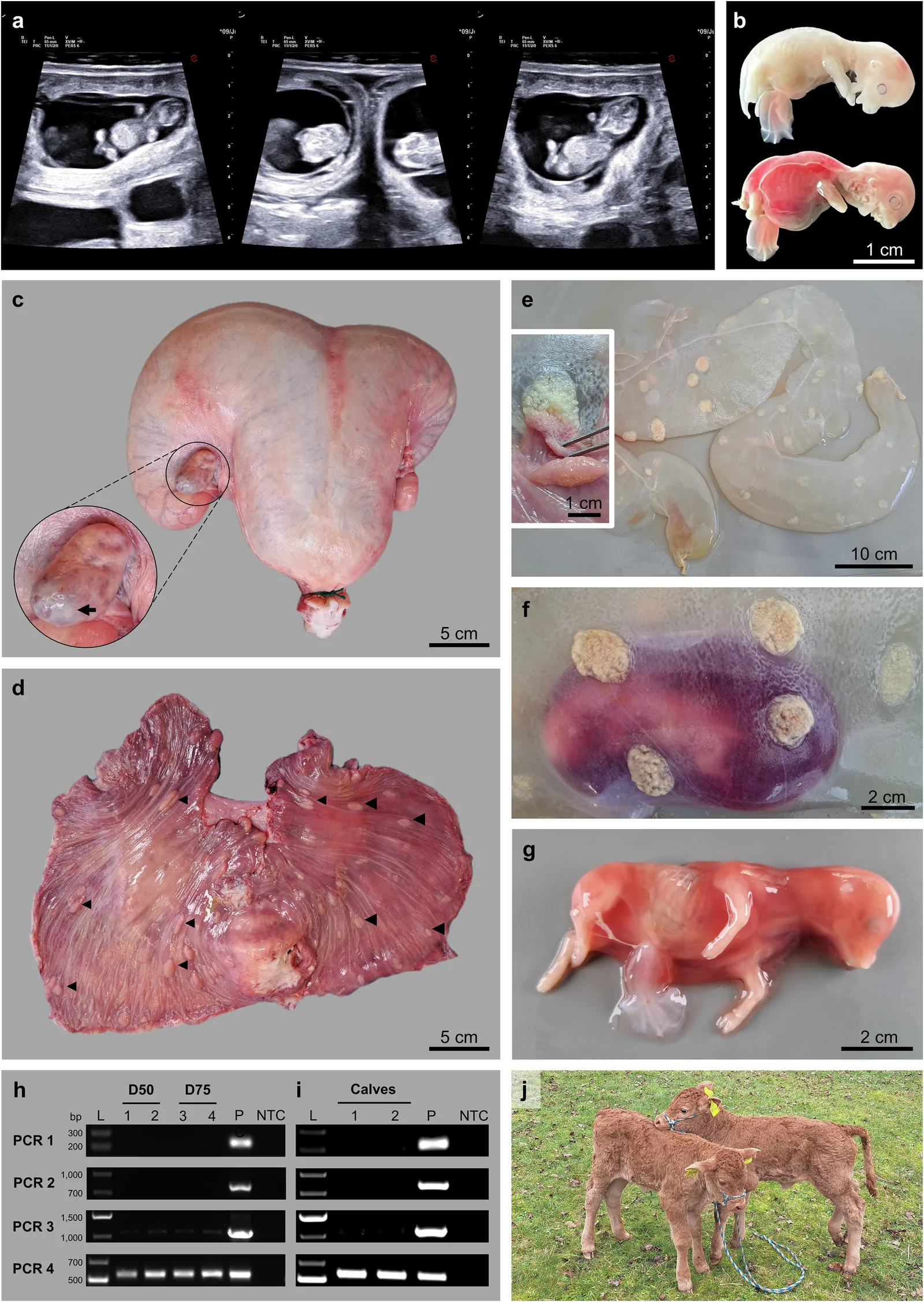
Establishment and progression of pregnancy in the absence of embryonic IFNT. **a** Transrectal ultrasonography (day 49) confirming a twin pregnancy in a recipient (67927) after transfer of two *IFNT*-KO SCNT embryos. **b** Fetuses recovered at day 50 of gestation following elective termination of another twin pregnancy (recipient 67942). One fetus appeared morphologically normal, while the second displayed features typical of SCNT-associated abnormalities. **c** Gross anatomy of the uterus and ovary recovered from recipient 67941 at day 75 of gestation, showing a normally developed uterus and a functional corpus luteum. **d** Distribution of maternal caruncles throughout the uterus following dissection. **e** Representative placentomes demonstrating normal attachment between maternal caruncles and fetal cotyledons. **f** Chorioallantoic membrane showing evenly distributed fetal cotyledons. **g** Fetus recovered at day 75 of gestation, exhibiting normal morphology without overt developmental abnormalities. A second fetus exhibiting cloning abnormalities is shown in Supplementary Fig. 5. **h** PCR-based genotyping of genomic DNA from fetuses recovered at days 50 and 75 (*n* = 2 independent biological replicates), confirming complete deletion of all *IFNT* loci. **i** PCR-based genotyping of genomic DNA from the distinct female calves (*n* = 2), demonstrating complete *IFNT* knockout. *GAPDH* served as an internal control. PCR-based genotyping presented in 4h and 4i was independently performed twice. **j** Representative photographs of the two healthy *IFNT*-null female calves born on February 26, 2026. Source data for **h** and **i** are provided in the Source Data file. KO, knockout; L, DNA ladder; NTC, no-template control; P, positive control.

Following the demonstration that *IFNT* ablation does not impair peri-implantation development, we next assessed whether *IFNT*-KO embryos could establish and sustain pregnancy. To this end, an independent series of embryo transfers was performed with SCNT-derived *IFNT*-KO blastocysts. Ten estrus-synchronized recipient heifers received either two (*n* = 8) or three (*n* = 2) *IFNT*-KO blastocysts per animal. Estrus behavior was monitored via automated neck-collar accelerometers and processed by the Lely Horizon management platform (Lely, Maassluis, Netherlands), generating time-series activity profiles to map estrus-associated behavioral dynamics (Supplementary Fig. 3). Two recipients (67914 and 67929) that received three embryos each returned to estrus within 20–21 days. In contrast, the remaining eight recipients (80%) exhibited prolonged luteal phases (Supplementary Table 2). Transrectal ultrasonography performed on days 40 (Supplementary Video 1), 47, and 49 post-estrus confirmed twin pregnancies in three recipients (Fig. 4a, and Supplementary Fig. 4).

One twin pregnancy was electively terminated at day 50 of gestation, yielding two fetuses, one of which appeared morphologically normal, while the second displayed features consistent with known cloning-associated developmental abnormalities (Fig. 4b). A second twin pregnancy was terminated at day 75 to assess placental development and maternal-fetal attachment. Gross anatomical examination revealed an appropriately sized uterus and a functional corpus luteum on the left ovary (Fig. 4c). Post-dissection analysis showed a well-distributed pattern of maternal caruncles throughout the endometrium, with approximately 60 distinct caruncles identified (Fig. 4d). Placentomes appeared morphologically normal, exhibiting tight, interdigitated attachment between the fetal cotyledons and maternal caruncles (Fig. 4e). Approximately 72 cotyledons were evenly distributed across the chorioallantoic membrane (Fig. 4f). One fetus recovered at day 75 exhibited normal morphology, with no overt developmental anomalies (Fig. 4g), while its twin showed structural alterations typical of SCNT-derived artifacts (Supplementary Fig. 5). PCR-based genotyping confirmed that all recovered fetuses, including those collected at days 50 and 75 of gestation, harbored complete deletions across all *IFNT* loci, matching the genotypes of their respective donor single-cell clone (Fig. 4h). The third twin pregnancy progressed successfully to term, culminating in the birth of two healthy female calves on February 26, 2026. Both calves were confirmed to be *IFNT*-null by genotyping (Fig. 4i, j) and showed physiological parameters within normal ranges (Supplementary Table 3).

To directly address whether a single *IFNT*-null conceptus is sufficient to establish and sustain pregnancy, we performed a subsequent round of transfers in which 4 synchronized recipients each received a single *IFNT*-KO blastocyst. Transrectal ultrasonography performed on day 36 of gestation confirmed a successful pregnancy in one recipient, which was subsequently reconfirmed on day 46 (Supplementary Fig. 6, Supplementary Video 2, and Supplementary Table 4), with the pregnancy remaining ongoing. This result definitively demonstrates that even a single *IFNT*-null conceptus is fully capable of preventing luteolysis and sustaining pregnancy, thereby ruling out the possibility that successful outcomes in the transfers with two blastocysts could be due to a dose-dependent effect of redundant secondary signals. Across all experimental cohorts, the overall ultrasound-confirmed pregnancy rate was 4/14 (28.6%), representing a remarkably high developmental efficiency for an SCNT-derived gene-knockout model.

## Discussion

MRP in ruminants has long been considered one of the most clearly defined endocrine paradigms in reproductive biology, with conceptus-derived IFNT viewed as the indispensable signal that prevents luteolysis and enables pregnancy establishment. This model was derived from decades of correlative, gain-of-function, and signaling studies, which concluded that IFNT inhibits the upregulation of uterine *ESR1* and *OXTR* expression, induces ISGs, and prolongs the lifespan of the corpus luteum^2–15,18,21–23^. Here, we fundamentally challenge this paradigm by demonstrating that embryonic IFNT is dispensable for the initiation, maintenance, and full-term progression of pregnancy in cattle.

To establish an uncompromised foundation for our conclusions, we achieved complete ablation of all annotated *IFNT* loci in BFFs. The absolute absence of a functional ligand was rigorously confirmed through a convergence of genomic, proteomic, and functional validations, including highly sensitive LC-MS/MS quantification targeting IFNT-specific proteotypic peptides. Notably, *IFNT*-KO embryos developed normally to the blastocyst stage in vitro and secreted no detectable IFNT during extended culture, yet successfully established pregnancies following transfer to synchronized recipients.

*IFNT*-KO conceptuses recovered at the peri-implantation stage (day 18) were morphologically indistinguishable from WT controls and exhibited comparable elongation. This finding contrasts with a previous report in sheep, where a morpholino antisense oligonucleotide targeting *IFNT* resulted in compromised conceptus elongation, suggesting an active autocrine role for IFNT during early development^6^. While species-specific differences remain a possibility, this phenotypic divergence is more likely explained by the profound technical distinctions between transient translational knockdowns and stable genetic knockouts. In mammalian embryology, morpholino-mediated knockdowns frequently introduce confounding off-target artifacts or cellular stress that do not emerge in clean genetic models^32^.

Our findings regarding the dispensability of autocrine IFNT signaling for conceptus elongation are strongly corroborated by a genetic model in sheep, in which embryos with a complete genomic knockout of *IFNAR2* developed normally and yielded viable rams^29^. Because IFNAR2 is thought to be mandatory for IFNT signaling, its ablation renders the developing embryo functionally blind to any autocrine effects of its own conceptus-derived IFNT.

Following the publication of our findings, Davies et al.^30^ reported that *IFNAR2*-KO ewes are fertile and can establish successful pregnancies through natural breeding, giving birth to viable lambs^30^. Notably, the parents and hence embryos in these pregnancies had intact *IFNT*. Thus, while this study further challenges the absolute requirement for IFNT-IFNAR signaling, it does not directly address the paracrine action of conceptus-derived IFNT on the maternal endometrium during MRP. An intriguing possibility could be the existence of an alternative IFNT receptor. By contrast, our genetic loss-of-function model in cattle provides the first direct, ligand-level confirmation that the IFNT-IFNAR signaling axis is entirely dispensable for pregnancy establishment and maintenance in ruminants. Nevertheless, generating a complementary ovine model with complete genetic ablation of *IFNT* remains essential to establish a definitive cross-species conclusion.

Surprisingly, quantitative global proteomics and transcriptomics of day-18 *IFNT*-KO and WT conceptuses revealed highly similar molecular profiles, with no evidence of upregulated alternative interferons or aberrant induction of collateral signaling pathways. These congruent molecular profiles underscore the high on-target efficiency and specificity of the sgRNAs utilized for *IFNT* ablation, confirming the lack of biologically relevant off-target effects. Moreover, the lack of intra-conceptus adaptation argues against a simple compensatory mechanism. Instead, it suggests the existence of a distinct, autonomous, and IFNT-independent pathway that operates natively in both KO and WT conceptuses to drive MRP, support conceptus elongation, and protect the corpus luteum.

A compelling mediator of this alternative embryo-maternal dialogue is an embryo-driven prostaglandin signaling cascade. Historically, intrauterine infusion of exogenous prostaglandin E_2_ (PGE_2_) alone has proven sufficient to extend luteal lifespan and mimic the presence of a conceptus in ewes^33^, heifers^34^, and gilts^35^. Mechanistically, the uterine and conceptus-derived PGE_2_ pool is actively transported via a local countercurrent mechanism through the utero-ovarian plexus (UOP) directly to the ovary^36^. Upon reaching the ovary, this influx acts as a critical signal that shifts the intraluteal PGE_2_ to PGF_2_α ratio away from luteolysis (PGF_2_α-dominant) and toward luteal preservation (PGE_2_-dominant)^36^.

Our targeted metabolomic and transcriptomic/proteomic analyses directly substantiate this hypothesis. PGE_2_ showed robust accumulation in day-18 peri-implantation conceptuses, with levels statistically indistinguishable between the *IFNT*-KO and WT cohorts. Concordantly, the primary terminal enzymes critical for PGE_2_ biosynthesis (PTGES, PTGES2, and PTGES3) were robustly and equivalently expressed across both cohorts. This expression signature aligns with conserved mammalian strategies, such as those observed in suids, where the early conceptus strongly upregulates key prostaglandin synthesis enzymes, including PTGES, to autonomously subvert maternal luteolytic signaling^37^. We therefore speculate that the elongating bovine conceptus utilizes this conserved prostaglandin signaling network to drive a localized luteoprotective environment, characterized by an elevated PGE_2_-to-PGF_2_α ratio. Nonetheless, comprehensive temporal profiling of embryonic PGE_2_ dynamics and formal genetic loss-of-function investigations remain essential to definitively establish its obligatory role during MRP in cattle.

While the absence of IFNT did not affect the conceptus molecular profile, the maternal response showed an absolute divergence between cohorts. Through its paracrine action on the endometrium, IFNT orchestrates the robust induction of an intricate maternal ISG network^38,39^. These downstream ISGs have served as pregnancy biomarkers and are known to orchestrate peri-implantation immunomodulation, antiviral defense, and endometrial receptivity^40–50^. In this study, the endometrium of recipients carrying WT embryos exhibited the expected IFNT-driven response, characterized by the robust upregulation of canonical ISGs (*ISG15*, *MX1*, *MX2*, *STAT1*, *STAT2*, and members of the *IFIT* family). In striking contrast, recipients carrying *IFNT*-KO embryos exhibited a complete absence of endometrial interferon signaling, with profound suppression of all IFNT-responsive genes and pathways. Remarkably, despite the complete absence of a maternal interferon signature, all recipients carrying *IFNT*-KO embryos evaluated at days 18, 50, and 75 maintained morphologically normal and functional corpora lutea. This sustained luteal support unambiguously demonstrates that luteolysis can be prevented independently of uterine IFNT signaling.

Although our initial embryo transfers utilized two blastocysts per recipient, one of which culminated in the birth of two live, clinically normal calves, we performed an additional round of single-embryo transfers to exclude any potential dose effect from multiple conceptuses. Transfer of a single *IFNT*-KO blastocyst successfully established a viable singleton pregnancy that continues to demonstrate normal gestational progression. Together, these findings definitively demonstrate that a single *IFNT*-null conceptus is fully sufficient to prevent luteolysis and support development, thereby ruling out a simple numerical dose effect of compensatory signals.

Nevertheless, IFNT may still play important modulatory roles during ruminant pregnancy. Because ruminant placentation does not permit transplacental transfer of maternal immune protection, embryonic IFNT may function as a conceptus-derived antiviral defense mechanism. This concept is supported by the evolution of IFNT-evasion strategies in bovine viral diarrhea virus, a major cause of infertility, early embryonic loss, and the birth of persistently infected offspring^51^. Although this potential role warrants further investigation, no evidence of viral infection was observed in any pregnancies that progressed beyond the peri-implantation stage in this study.

From a translational perspective, our findings prompt a fundamental paradigm shift in how we approach and treat infertility in ruminants. For decades, diagnostic strategies and therapeutic interventions aimed at mitigating early pregnancy loss have focused almost exclusively on monitoring or supplementing IFNT signaling. Our results demonstrate that this historical focus targets a non-obligatory, parallel pathway rather than the real driver of pregnancy recognition. By demonstrating that early gestation can be established and maintained beyond key developmental milestones in the complete absence of a maternal interferon response, these data reveal that the ruminant embryo relies on a distinct, autonomous, and self-sufficient signaling axis to override the maternal luteolytic clock. Consequently, the IFNT-deficient model developed in this study provides a powerful platform for identifying and validating alternative maternal recognition pathways in domestic livestock and for developing novel strategies to improve reproductive efficiency.

## Methods

### Ethical approval and animal use

All animal procedures were conducted in accordance with the German Animal Welfare Act (Tierschutzgesetz) and with permission from the responsible authority, the Government of Upper Bavaria (ROB-55.2-2532.Vet_02-24-131). Bovine ovaries were obtained from a slaughterhouse (Münchner Schlachthof Betriebs GmbH, Munich, Germany). In vitro produced embryos were obtained from an EU-approved bovine embryo collection and production center at the Chair for Molecular Animal Breeding and Biotechnology of the LMU Munich (Moorversuchsgut Badersfeld, Oberschleißheim, Germany; approval number DE ETR 006 EWG).

Animal experiments utilized cattle (*Bos taurus*). Primary nuclear donor cells (BFF-091019) were established from an early-gestation female Limousin fetus (*B. taurus*), and all generated embryos, day-18 conceptuses (*n* = 4 *IFNT*-null, *n* = 3 wild-type), day-50 fetuses (*n* = 2 *IFNT*-null), day-75 fetuses (*n* = 2 *IFNT*-null), and live-born calves (*n* = 2 *IFNT*-null) were female. Sex was not evaluated as a comparative biological variable in the study design or statistical analyses, and sex-disaggregated analysis within or between experimental groups was not applicable. A total of 22 cyclic recipient heifers, primarily of the German Simmental breed (*B. taurus*) and crossbreds (German Simmental × Holstein-Friesian), were utilized for estrus synchronization and embryo transfers.

### Generation of *IFNT*-KO cells

Primary BFF cells, internally designated as BFF-091019, were established from an early-gestation female Limousine fetus (*B. taurus*) obtained from the slaughterhouse following standard protocol. Cells were maintained under standard culture conditions at 37 °C with controlled humidity and 5% CO₂ in fibroblast growth medium supplemented with 15% fetal calf serum (FCS), non-essential amino acids, and HEPES buffer. The single-guide RNA (sgRNA) design and genomic locus annotation were based on the latest ARS-UCD2.0 bovine genome assembly (NCBI RefSeq assembly GCF_002263795.3; GenBank assembly GCA_002263795.4) submitted by Rosen et al.^52,53^. The targeted region included all six alleles of the three functional *IFNT* genes: *IFN-tau-c1* (Gene ID: 528153), *IFNT2* (Gene ID: 317698), and *IFNT3* (Gene ID: 100313956). Multiple sgRNAs targeting all annotated functional *IFNT* loci were designed by using established in silico tools to ensure high on-target efficiency and minimal predicted off-target effects. Several sgRNA pairs were tested, and the pair demonstrating the highest editing efficiency across all *IFNT* copies (sgRNA1: catcagtagagagagcacga; sgRNA2: agtgcagagctgctccagga) was selected to generate the final *IFNT*-knockout (KO) donor cells for SCNT.

For genome editing, pre-assembled Cas9 ribonucleoprotein (RNP) complexes were prepared by mixing TrueCut™ Cas9 Protein v2 (Thermo Fisher Scientific, Waltham, MA, USA) with the selected sgRNAs, and subsequently delivered into BFFs by electroporation with the Amaxa Nucleofector I device. Edited cell populations were allowed to recover for 48–72 h before single-cell sorting and clonal expansion in 96-well plates. Clones were propagated, and genomic DNA (gDNA) was extracted for molecular characterization. Three independent PCR assays targeting distinct regions of the multigene *IFNT* locus were used to identify candidate knockout clones. Primer Pair 1 amplified a ~200-bp fragment of gDNA within the *IFNT* coding sequence (CDS) between the two sgRNA cut sites (CD-Forward: tgaggaccacatgctaggtg; CD-Reverse: ggaagaggttgaagcactgc). Primer Pair 2 spanned the gDNA from a forward primer overlapping the sgRNA1 cut site to a reverse primer in the 3′-UTR, yielding a ~774-bp product (CS1-Forward: gtgctctctctactgatggcc; 3′-Reverse: tcagggcaggcatcacttaa). Primer Pair 3 amplified a ~1045-bp gDNA fragment from the 5′-UTR to a reverse primer overlapping the sgRNA2 cut site (5′-Forward: gtgtattccccctccctgag; CS2-Reverse: gtgcagagctgctccagga). *GAPDH* served as a housekeeping control (F: cgggtgcaattaacttgctcctt; R: cttctgggtggcagtgatgg). Clones lacking amplification across all three *IFNT-*specific assays, indicative of complete deletion of all *IFNT* copies, while retaining *GAPDH* product, were designated as complete knockouts. Starting with the single parental BFF-091019 population, multiple single-cell clones were generated and screened for complete *IFNT*-KO. Following comprehensive molecular validation, two independent *IFNT*-KO single-cell clones (SCC-22 and SCC-34) were selected for all subsequent in vitro functional assays and in vivo embryo transfer experiments on the basis of their confirmed complete deletion of all functional *IFNT* alleles and superior growth characteristics, including morphology and proliferation rate. Unedited parental BFF-091019 cells served as WT controls throughout genotyping and SCNT procedures.

### Oocyte maturation for SCNT and IVF

Unless otherwise indicated, chemicals, media, and reagents used in experiments were purchased from Sigma Chemical Co. (St. Louis, MO, USA). Ovaries were collected from Brown Swiss and Simmental cows at a slaughterhouse and transported in phosphate-buffered saline (PBS) at 25–30 °C. Cumulus-oocyte complexes (COCs) were obtained by follicle aspiration, washed in TCM 199 Hepes, and transferred into four-well plates (Nunc, Life Technologies GmbH, Darmstadt, Germany) with 400 μL modified Parkers medium (MPM) containing TCM199 supplemented with L-glutamine (100 mg/L), NaHCO_3_ (800 mg/L), Hepes (1400 mg/L), sodium-pyruvate (250 mg/L), L-lactic-calcium-salt (600 mg/L), gentamicin (55 mg/L), and 5% estrous cow serum (ECS) containing 0.01 U/mL b-FSH and b-LH (Sioux Biochem, Sioux Center, IA, USA). For SCNT, oocytes were matured for 16–18 h at 39 °C in a humidified atmosphere of 5% CO₂ in air. Following maturation, cumulus cells were removed by incubation for 5 min in modified PBS (mPBS; PBS supplemented with 4 mg mL⁻¹ bovine serum albumin) containing 3 mg mL⁻¹ hyaluronidase, followed by vortexing for 4 min and gentle pipetting. For in vitro fertilization (IVF), oocytes were matured for 20–22 h under identical temperature and atmospheric conditions.

### Somatic cell nuclear transfer

SCNT was performed at 20–25 °C by using Leitz micromanipulators (Leica Microsystems, Wetzlar, Germany) mounted on a Wilovert stereo microscope. Denuded oocytes with a visible polar body were incubated for 1–2 h in TCM-199 Hepes supplemented with 10% FCS (Gibco, Life Technologies GmbH, Darmstadt, Germany) and 0.6 μg/mL demecolcine prior to enucleation^54^. The metaphase II spindle-chromatin complex was removed in a small drop of mPBS with an enucleation pipette. Enucleated oocytes were maintained in TCM-199 Hepes with 10% FCS and used as recipient cytoplasts. A single donor cell was placed beneath the zona pellucida of each enucleated oocyte. Karyoplast-cytoplast complexes (KCCs) were aligned between two 200-μm wire electrodes in Eppendorf fusion medium and fused by using an Eppendorf Multiporator (Hamburg, Germany) with two direct-current pulses of 1.95 kV/cm for 25 μs. After a 30-min recovery in TCM-199 Hepes with 10% FCS, fused KCCs were activated by a 5-min treatment with 7% ethanol, followed by 5 h in 10 μg/mL cycloheximide and 5 μg/mL cytochalasin B. Activated embryos were washed three times and transferred to 450-μL drops of synthetic oviduct fluid (SOF) supplemented with 2% basal medium Eagle (BME) amino acids, 1% MEM nonessential amino acids, and 5% (v/v) ECS. Culture drops were covered with paraffin oil (Merck, Darmstadt, Germany), and embryos were incubated at 39 °C in a humidified atmosphere of 5% CO₂, 5% O₂, and 90% N₂.

### In vitro fertilization and culture of embryos

Matured COCs were washed three times in fertilization medium (Tyrode’s albumin lactate pyruvate; FERT-TALP) supplemented with sodium pyruvate (2.2 mg/mL), heparin sodium salt (2 mg/mL), and BSA (6 mg/mL). The COCs were then transferred to 400-μL droplets of fertilization medium. Frozen-thawed spermatozoa, previously subjected to a 90-min swim-up procedure, were co-incubated with the COCs at a final concentration of 2 × 10⁶ cells/mL for 18 h at 39 °C in a humidified atmosphere containing 5% CO₂ in air. Presumptive zygotes were mechanically denuded by vortexing, washed three times in SOF culture medium supplemented with 2% BME amino acids, 1% MEM nonessential amino acids, and 5% (v/v) ECS, and transferred to 400-μL droplets of culture medium covered with mineral oil. Embryos were cultured at 39 °C under maximum humidity in an atmosphere containing 5% CO₂, 5% O₂, and 90% N₂.

### Long-term culture of bovine embryos

To analyze the release of IFNT by the embryos, long-term in vitro culture of embryos was performed following our previously published protocol^55,56^. Briefly, day-8 SCNT or IVF blastocysts were individually placed into 60-µL drops of Ham’s F12 medium supplemented with 10^−4 ^M 2-mercaptoethanol (Merck, Darmstadt, Germany) and 15% FCS. Culture was carried out at 37 °C in humidified air with 10% CO_2_. On Days 11, 15, 19, and 21, 30 µL of medium from each drop was collected for IFNT quantification, and the same volume of fresh culture medium was added to replace it. Samples were stored individually at −80 °C until further analysis. Because each embryo was cultured individually, the measured IFNT concentrations in the conditioned medium directly represent secretion per embryo.

### Western blot analysis

To confirm the *IFNT* knockout at the protein level, conditioned media from KO embryos and WT embryos (both from SCNT and IVF) were used. Western blots were also performed with protein extracts from day-18 KO and WT (SCNT) conceptuses. For IFNT protein detection, 10 μL of embryo-conditioned medium or 20 μg of protein from day-18 conceptuses was used. Samples were mixed with 2× Laemmli buffer containing 100 mM DTT and then separated on SDS-PAGE gels. Electrophoresis was performed in SDS-running buffer at 80 V until the samples entered the separating gel, and then at 100 V until complete separation was achieved. Total protein was visualized under UV via the Intas imaging system (Intas SII GmbH, Göttingen, Germany) to confirm equal loading. Proteins were transferred to nitrocellulose membranes with a wet transfer system (Bio-Rad Laboratories, Hercules, CA, USA) at 0.15 A for 75 min. Transfer efficiency was verified by UV imaging. Membranes were blocked with 5% (w/v) milk in TBS-T for 1 h at room temperature and incubated overnight at 4 °C with rabbit anti-bovine IFNT polyclonal antibody (Abbexa Ltd, Cambridge, UK; cat. no. abx177051) diluted 1:100,000 for conceptus extracts or 1:50,000 for embryo-conditioned media in 2% milk/TBS-T. After two 5-min washes with TBS-T, membranes were incubated for 1 h at room temperature with HRP-conjugated anti-rabbit secondary antibody (1:10,000; Cell Signaling Technology, Leiden, Netherlands; cat. no. 7074), followed by three washes (2 × 5 min, 1 × 10 min) with TBS-T. Proteins were detected with ECL Pico substrate (Thermo Fisher Scientific, Waltham, MA, USA) for 1 min in a light-protected container, and membranes were imaged with the Intas system. For loading control, anti-actin primary antibody (1:10,000; MP Biomedicals, Irvine, CA, USA; cat. no. 691001) and HRP-conjugated anti-mouse secondary antibody (1:10,000; Cell Signaling Technology, Leiden, Netherlands; cat. no. 7076) were used.

### Cytopathic effect inhibition assay to detect IFNT activity

To confirm the complete absence of IFNT at the functional level, embryo-conditioned media from the KO and WT (IVF) embryos were evaluated. Interferon activity in embryo-conditioned media was quantified by a classical viral cytopathic-effect (CPE) inhibition assay^57^, which measures the capacity of interferons to protect susceptible cells from virus-induced lysis. The assay was performed with Madin-Darby bovine kidney (MDBK; ATCC CRL-6071) cells challenged with vesicular stomatitis virus (VSV, Indiana strain). Briefly, recently split MDBK cells were incubated for 24 h with serial dilutions (50 µL each) of embryo-conditioned media, starting at 1:10 and proceeding in duplicate through eight twofold dilutions to a final dilution of 1:1280. Each 96-well test plate included a recombinant bovine IFNT reference standard and virus-only cytopathic effect controls. The VSV input dose was empirically calibrated to achieve complete CPE in untreated MDBK cultures. At this virus concentration, a checkerboard titration with recombinant IFNT (starting at 50.2 ng) was performed to establish the assay’s operational dynamic range. The concentration of recombinant IFNT required to achieve 50% inhibition of VSV-induced CPE (half-maximal effective concentration; EC₅₀) was determined to be 0.26 pg/µL, and this value was used to calculate interferon activity in experimental samples. As previously reported, antiviral activity in IVF embryo supernatants reflects the secretion of IFNT^55^. Interferon activity was expressed as the reciprocal of the highest dilution providing ≥50% protection, and samples exceeding the upper detection limit (i.e., protection maintained at the highest dilution tested) were reported as “> upper detection limit (UDL)”. Data were interpreted according to established analytical frameworks for antiviral interferon bioassays, allowing for a direct comparison of secreted IFNT activity between SCNT-derived *IFNT*-KO embryos and WT IVF embryos.

### Estrus synchronization of recipient heifers

Cyclic heifers, mostly of the German Simmental breed and some crossbred heifers from German Simmental and Holstein-Friesian, aged between 15 and 18 months and weighing 350 to 400 kg, were housed together as a group in a straw-bedded barn. Estrus synchronization was achieved by two intramuscular injections of the prostaglandin F2α analog cloprostenol (Estrumate®; Intervet, Unterschleißheim, Germany, 2 mL) administered 11 days apart. All animals exhibited estrus within 60 to 96 h after both the first and the second prostaglandin treatments.

### Embryo transfer and tissue collection

In the first round of embryo transfers for day-18 conceptus collections, two groups of synchronized recipients were established, KO and WT, each comprising four animals. In the WT group, recipients received either one (*n* = 1) or two (*n* = 3) WT SCNT blastocysts, whereas all recipients in the KO group received two *IFNT*-KO SCNT blastocysts each (*n* = 4). SOF medium containing 5% ECS with embryos was loaded into 0.25-mL straws. Before the transfer, animals were restrained in a standing position and administered a local epidural anesthesia with procaine hydrochloride (Procamidor^®^; Richter Pharma AG, Wels, Austria; 4 mL). The genital tract was cleaned under hygienic conditions, and embryos were deposited into the uterine horn ipsilateral to the functional corpus luteum via a standard transcervical transfer catheter. At 18 days of gestational age (dGA), terminal surgeries were performed on all recipients. Conceptuses were flushed from the uterus with D-PBS supplemented with 0.4% BSA, length was recorded, and tissues were snap-frozen. Uterine flushes were stored for subsequent proteomic analysis. Endometrial biopsies were collected from different locations in each uterine horn and washed for 5 s in a buffer (0.4 M Na_2_HPO_4_, 0.1 M NaH_2_PO_4_, 0.5 M NaCl) containing 10 IU/mL heparin, followed by snap freezing.

To evaluate whether genetic ablation of *IFNT* affects pregnancy establishment beyond the peri-implantation period, we performed two independent rounds of embryo transfers. In the first round, ten synchronized recipients received either two (*n* = 8) or three (*n* = 2) *IFNT*-KO embryos per animal. Return to estrus was monitored by using automated neck-collar accelerometers and processed by the Lely Horizon management platform (Lely, Maassluis, Netherlands), whereas pregnancy progression was monitored by transrectal ultrasonography (MyLab™ Heron ultrasound device equipped with an SV 3513 linear probe; Esaote VET, Genoa, Italy). Two pregnant recipients carrying *IFNT*-KO embryos were electively euthanized at gestational days 50 and 75, respectively. Euthanasia was performed by intravenous administration of pentobarbital (Release^®^; WDT, Garbsen, Germany; 100 mL).

An additional round of transfers was performed with four synchronized recipients, each receiving a single *IFNT*-KO blastocyst. One recipient successfully established a viable singleton pregnancy that was confirmed by ultrasonography on days 36 and 46 and remains ongoing.

### RNA isolation and sequencing

The frozen conceptus tissue was immersed in 1 mL TRIzol reagent (Invitrogen) supplemented with 20 µL thioglycerol and homogenized. A 200-µL aliquot of the TRIzol lysate was processed for RNA isolation by using the Maxwell RSC 48 instrument and the Maxwell RSC miRNA Tissue Kit (Promega), according to the manufacturer’s instructions. RNA concentration and purity were assessed by a NanoDrop spectrophotometer, and RNA integrity was evaluated with a Bioanalyzer (Agilent Technologies, Santa Clara, CA, USA). Only samples with high RNA integrity (RIN > 8.0) were used for library preparation. A total of 500 ng RNA per sample was used to generate full-length cDNA libraries with the Oxford Nanopore Technologies cDNA PCR Barcoding Kit, following the manufacturer’s protocol. Barcoded libraries were quantified, pooled at equimolar concentrations, and sequenced on a PromethION P24 platform (Oxford Nanopore Technologies, Oxford, UK) by using FLO-PRO114M flow cells for 72 h.

Basecalling was performed with Dorado (v1.4.0) by using the super-accurate (sup) model. Residual adapter sequences were removed with Porechop (v0.2.4). Trimmed reads were aligned to the *Bos taurus* ARS-UCD2.0 reference genome by Minimap2 (v2.30-r1287) with the -x splice:hq parameter. Transcript quantification and generation of gene-level counts were performed by IsoQuant (v3.12.2) with the NCBI *Bos taurus* reference annotation as a guide. Differential gene expression analysis was conducted in R (v4.5.0) with the edgeR package (v4.8.2). Raw counts were filtered to remove lowly expressed genes by using the filterByExpr function. Library sizes were normalized with the trimmed mean of M-values (TMM) method. Dispersion was estimated, and differential expression was tested with a quasi-likelihood (QL) negative binomial generalized log-linear model.

### Sample preparation and LC/ESI-MS/MS analysis of prostaglandins

Approximately 20 mg of tissue was homogenized and spiked with a mixture of deuterated internal standards (100 pg each): PGB₂-d₄, PGE₂-d₄, PGE₂−13,14-dihydro-15-keto-d₄, PGF₂α-d₄, PGJ₂-d₄, and 15-deoxy-Δ¹²,¹⁴-PGJ₂-d₄ (Cayman Chemical, Ann Arbor, USA). Methanol was added for protein precipitation. After centrifugation, the supernatant was adjusted to pH 6.0 and subjected to solid-phase extraction on Bond Elut Certify II columns (Agilent Technologies). The eluate was evaporated to dryness in a SpeedVac concentrator (Thermo Electron, Waltham, MA, USA), and the residue was reconstituted in 100 µL methanol/water (1:1, v/v).

Prostaglandins were analyzed by using an Agilent 1290 Infinity HPLC system equipped with a binary pump, multisampler, and column thermostat, coupled to an Agilent 6495 triple quadrupole mass spectrometer with an electrospray ionization (ESI) source. Chromatographic separation was performed on a Zorbax Eclipse Plus C18 column (2.1 × 150 mm, 1.8 µm; Agilent Technologies) maintained at 40 °C. The mobile phase consisted of 0.05% acetic acid in water (solvent A) and acetonitrile (solvent B). Analytes were eluted at 0.3 mL/min using a linear gradient, with an injection volume of 20 µL. Mass spectrometric detection was performed in negative-ion mode with multiple reaction monitoring. At least two specific mass transitions were monitored for each prostaglandin and its corresponding internal standard. Data were acquired and processed via Agilent MassHunter software.

### Sample preparation and LC-MS/MS proteomic analysis

To remove cell debris, uterine flush solutions were centrifuged at 3200 × *g* for 15 min at 4 °C. 0.1 M NH_4_HCO_3_ solution was added to the samples to a final concentration of ~50 mM NH_4_HCO_3_. Endometrial tissue samples and trophectoderm samples were lysed by the addition of 8 M urea, 0.4 M NH_4_HCO_3_, and ultrasonicated with 24 ×10 s pulses (Bandelin Sonopuls UW3200, Bandelin, Berlin, Germany). The lysates were centrifuged at 20,817 × *g* for 15 min at 4 °C, and the supernatant was recovered. Protein quantification was performed with Pierce 660 nm Protein Assay (Thermo Fisher Scientific, Rockford, IL, USA). Proteins were reduced by adding dithiothreitol to a final concentration of 5 mM and incubating at 37 °C for 30 min. Subsequently, alkylation was performed by adding iodacetamide to a final concentration of 15 mM and incubating in the dark at room temperature for 30 min. Digestion was performed at 37 °C for 4 h by using Lys-C (FUJIFILM Wako Chemicals Europe GmbH, Neuss, Germany) in a protease-to-sample ratio of 1:100. For the endometrium and trophectoderm samples, the final concentration was diluted with water to 1 M urea. Digestion was completed by adding modified porcine trypsin (Promega, Madison, WI, USA) in a protease-to-sample ratio of 1:50, followed by overnight incubation at 37 °C. The digestion was stopped by adding formic acid to a final concentration of 1%.

A nanoElute 2 LC system, coupled with a timsTOF HT mass spectrometer (both from Bruker Daltonics, Bremen, Germany), was used for mass spectrometry analysis. For each sample, 400 ng of peptides were injected on a trap column (Pepmap NEO C18, 300 µm × 5 mm, ThermoScientific) and separated at a flow rate of 250 nL/min on a PepSep column (25 cm × 75 µm, IonOpticks), with the following eluents: Eluent A was 0.1% formic acid in water and B was 0.1% formic acid in acetonitrile. The separation method consisted of an initial ramp from 2% to 25% eluent B over 25 min, followed by a 12-min gradient to 37%. MS spectra were acquired in dia-PASEF mode with 21–25 *m/z* wide windows and an ion mobility range of 0.85–1.27 k/e. Protein identification was performed with DIA-NN (v2.1.0)^58^ and the *Bos taurus* reference proteome (Proteome identifier UP000009136, last accessed on 28 Aug 2025) at UniProt.org. Only tryptic peptides with ≤1 missed cleavage, a peptide length of 7-30 amino acids, and charge states from +2 to +4 were considered. As fixed modification cysteine carbamidomethylation was set, and DIA-NN’s Robust LC high-accuracy mode with automatic retention-time correction was used. Mass tolerances were set automatically. Protein counts were derived from the “Genes” column, with all other parameters left at default. Precursor-level output (report.parquet) was processed in R (v4.5.1) and filtered at 1% FDR using global and run-specific precursor *q* values and global protein-group *q* values.

Data analysis and statistical evaluation were performed via Perseus (v2.1.5)^59^. Proteins exhibiting reduced abundance in endometrial samples from *IFNT*-KO embryos were further analyzed for pathway enrichment by using STRING (string-db.org), with Reactome as the reference pathway database. For IFNT quantification, multiple peptides were considered, and only peptides that were proteotypic for at least one interferon tau subtype were retained for analysis to ensure the specificity of IFNT detection and quantification.

### Statistics and reproducibility

No statistical method was used to predetermine sample size. Sample sizes were selected based on established standards in bovine SCNT and embryology. No data were excluded from the analyses. The experiments were not randomized. For in vitro assays and sample evaluations (including embryo-conditioned media analyses), investigators were blinded to sample identity and treatment groups during outcome assessment. For in vivo procedures, investigators were not blinded to allocation during experiments. All statistical analyses and generation of developmental competence and bioassay graphs were performed using GraphPad Prism (v10.6.1, GraphPad Software, Boston, MA, USA) as described in the figure legends. Sample sizes (*n*) represent biological replicates. Representative PCR gels and western blots reflect findings from independent biological replicates or were independently repeated at least twice with consistent results. Uncropped images of all gels and blots, along with numerical data for the graphs, are provided in the Source Data file.

### Reporting summary

Further information on research design is available in the Nature Portfolio Reporting Summary linked to this article.

## Supplementary information

Supplementary Information

Description of Additional Supplementary Files

Supplementary Data 1

Supplementary Data 2

Supplementary Data 3

Supplementary Data 4

Supplementary Data 5

Supplementary Data 6

Supplementary Data 7

Supplementary Video 1

Supplementary Video 2

Reporting Summary

Transparent Peer Review file

## Source data

Source Data

## Data Availability

The proteomics data generated in this study have been deposited in the MassIVE repository under accession code MSV000100363 and are accessible via ProteomeXchange under accession code PXD072556. The RNA sequencing data have been deposited in the European Nucleotide Archive (ENA) under BioProject accession code PRJEB125247. The reference genome assembly used in this study is ARS-UCD2.0 [https://www.ncbi.nlm.nih.gov/assembly/GCF_002263795.3/]. The reference proteome dataset used is UP000009136. Source data are provided with this paper. No custom code or algorithms were generated or used in this study. All analyses were conducted using standard, publicly available software packages as described in the Methods. Source data are provided with this paper.
