# Supplementary material for: Pregnancy establishment in cattle without embryonic interferon tau
Source: Nat Commun. 2026 Oct 8;17:10609. doi: 10.1038/s41467-026-78446-4 (PMC13645731; doi:10.1038/s41467-026-78446-4)
Supplement: Supplementary file 1 [file 41467_2026_78446_MOESM12_ESM.pdf]

nature portfolio

Corresponding author(s): Asghar Ali
Eckhard Wolf

Last updated by author(s): Sep 8, 2026

Reporting Summary

Nature Portfolio wishes to improve the reproducibility of the work that we publish. This form provides structure for consistency and transparency in reporting. For further information on Nature Portfolio policies, see our Editorial Policies and the Editorial Policy Checklist.

Statistics

For all statistical analyses, confirm that the following items are present in the figure legend, table legend, main text, or Methods section.

n/a	Confirmed	
☐	☒	The exact sample size (*n*) for each experimental group/condition, given as a discrete number and unit of measurement
☐	☒	A statement on whether measurements were taken from distinct samples or whether the same sample was measured repeatedly
☐	☒	The statistical test(s) used AND whether they are one- or two-sided<br>*Only common tests should be described solely by name; describe more complex techniques in the Methods section.*
☒	☐	A description of all covariates tested
☐	☒	A description of any assumptions or corrections, such as tests of normality and adjustment for multiple comparisons
☐	☒	A full description of the statistical parameters including central tendency (e.g. means) or other basic estimates (e.g. regression coefficient) AND variation (e.g. standard deviation) or associated estimates of uncertainty (e.g. confidence intervals)
☐	☒	For null hypothesis testing, the test statistic (e.g. *F*, *t*, *r*) with confidence intervals, effect sizes, degrees of freedom and *P* value noted<br>*Give P values as exact values whenever suitable.*
☒	☐	For Bayesian analysis, information on the choice of priors and Markov chain Monte Carlo settings
☒	☐	For hierarchical and complex designs, identification of the appropriate level for tests and full reporting of outcomes
☒	☐	Estimates of effect sizes (e.g. Cohen's *d*, Pearson's *r*), indicating how they were calculated

Our web collection on statistics for biologists contains articles on many of the points above.

Software and code

Policy information about availability of computer code

Data collection	No custom code was used for data collection. Standard commercial software bundled with the respective instruments (e.g., mass spectrometry acquisition software and high-throughput sequencer control software) was used for data acquisition.
Data analysis	Transcriptomic Analysis: Dorado (v1.4.0), Porechop (v0.2.4), Minimap2 (v2.30-r1287), IsoQuant (v3.12.2), and edgeR (v4.8.2) in R (v4.5.0) were used for Oxford Nanopore cDNA sequencing processing, basecalling, read alignment, transcript quantification, and differential gene expression analysis.Proteomic Analysis: DIA-NN (v2.1.0) and Perseus (v2.1.5) were used for dia-PASEF LC-MS/MS data processing, protein identification, and quantitative analysis against the Bos taurus reference proteome. Pathway enrichment analysis was performed using STRING with the Reactome database.Targeted Metabolomics: Agilent MassHunter software was used for LC/ESI-MS/MS data acquisition and processing of prostaglandin profiles.Statistical Analysis & Visualization: R (v4.5.1) and GraphPad Prism (v10.6.1) were used for statistical testing and figure generation. No custom algorithms or custom code were generated or used in this study.

For manuscripts utilizing custom algorithms or software that are central to the research but not yet described in published literature, software must be made available to editors and reviewers. We strongly encourage code deposition in a community repository (e.g. GitHub). See the Nature Portfolio guidelines for submitting code & software for further information.

Data

Policy information about availability of data

All manuscripts must include a data availability statement. This statement should provide the following information, where applicable:

- Accession codes, unique identifiers, or web links for publicly available datasets
- A description of any restrictions on data availability
- For clinical datasets or third party data, please ensure that the statement adheres to our policy

The proteomics data generated in this study have been deposited in the MassIVE repository under accession code MSV000100363 [https://massive.ucsd.edu/ProteoSAFe/dataset.jsp?task=MSV000100363] and are accessible via ProteomeXchange under accession code PXD072556 [http://proteomecentral.proteomexchange.org/cgi/GetDataset?ID=PXD072556]. The RNA sequencing data have been deposited in the European Nucleotide Archive (ENA) under BioProject accession code PRJEB125247 [https://www.ebi.ac.uk/ena/browser/view/PRJEB125247]. The reference genome assembly used in this study is ARS-UCD2.0 [https://www.ncbi.nlm.nih.gov/assembly/GCF_002263795.3/]. The reference proteome dataset used is UP000009136 [https://www.uniprot.org/proteomes/UP000009136]. Source data are provided with this paper. No custom code or algorithms were generated or used in this study. All analyses were conducted using standard, publicly available software packages as described in the Methods.

Research involving human participants, their data, or biological material

Policy information about studies with human participants or human data. See also policy information about sex, gender (identity/presentation), and sexual orientation and race, ethnicity and racism.

Reporting on sex and gender	n/a
Reporting on race, ethnicity, or other socially relevant groupings	n/a
Population characteristics	n/a
Recruitment	n/a
Ethics oversight	n/a

Note that full information on the approval of the study protocol must also be provided in the manuscript.

Field-specific reporting

Please select the one below that is the best fit for your research. If you are not sure, read the appropriate sections before making your selection.

☒ Life sciences ☐ Behavioural & social sciences ☐ Ecological, evolutionary & environmental sciences

For a reference copy of the document with all sections, see nature.com/documents/nr-reporting-summary-flat.pdf

Life sciences study design

All studies must disclose on these points even when the disclosure is negative.

Sample size	Sample sizes were determined based on established standards for large-animal somatic cell nuclear transfer (SCNT) and embryo transfer studies rather than pre-experiment statistical power calculations. To ensure biological reproducibility across all developmental milestones, adequate numbers of SCNT-derived blastocysts, recipient animals (including single-embryo transfer models), day-18 conceptuses, and tissue biopsies were utilized, as detailed in the Animal details and Methods sections. For all experimental cohorts subjected to statistical testing, a minimum of three independent biological replicates ($n \geq 3$) was analyzed per group.
Data exclusions	No data or samples were excluded from the analysis.
Replication	All experimental attempts at replication were successful. Key findings—including the establishment of pregnancy in the absence of IFNT—were replicated across independent embryo transfer sessions and multiple recipient animals. Key PCRs and Western blots were also repeated at least twice. All assays included multiple samples from independent biological replicates, and produced consistent results.
Randomization	Recipient cattle were randomly assigned to receive either wild-type (WT) or IFNT-null SCNT embryos. Baseline covariates, including recipient age, health status, and estrous cycle synchronization timing, were balanced across experimental groups.
Blinding	Investigators conducting in vitro downstream laboratory assays (qPCR, RNA-seq transcriptomics, LC-MS/MS proteomics, and endometrial biopsy evaluations) were blinded to sample group allocations during data acquisition and analysis. Blinding during embryo reconstruction and transfer was not possible due to strict genotype tracking protocols.

Reporting for specific materials, systems and methods

We require information from authors about some types of materials, experimental systems and methods used in many studies. Here, indicate whether each material, system or method listed is relevant to your study. If you are not sure if a list item applies to your research, read the appropriate section before selecting a response.

Materials & experimental systems

n/a	Involved in the study
☐	☒ Antibodies
☐	☒ Eukaryotic cell lines
☒	☐ Palaeontology and archaeology
☐	☒ Animals and other organisms
☒	☐ Clinical data
☒	☐ Dual use research of concern
☒	☐ Plants

Methods

n/a	Involved in the study
☒	☐ ChIP-seq
☒	☐ Flow cytometry
☒	☐ MRI-based neuroimaging

Antibodies

Antibodies used

Interferon Tau-1 (IFNT1) Antibody;
Cat No. abx177051; Immunogen, abx652175 - Recombinant Cow IFNT1 (Cys24-Leu195)
Dilution: 1:50,000 to 1:100,000

anti-Actin antibody
Clone: C4, Supplier: FischerScientific/MP Biomedicals
Catalog number: ICN691001
Dilution: 1:10,000

Validation

IFNT antibody was validated in our study by clear knockout experiments.
Also previously validated in DOI: 10.1615/CritRevEukaryotGeneExpr.2023047178.

Anti-ACTN antibody from MP Biomedicals is a validated housekeeping/loading control, and has been cited numerous times.

Eukaryotic cell lines

Policy information about cell lines and Sex and Gender in Research

Cell line source(s)

Primary bovine fetal fibroblasts (BFFs) were established in-house from female Limousine (Bos taurus) fetus.

Authentication

Cell lines were authenticated by PCR, Sanger sequencing, and deep targeted genomic sequencing to confirm complete CRISPR-Cas9 deletion across the IFNT multigene locus.

Mycoplasma contamination

All cell lines were negative for Mycoplasma.

Commonly misidentified lines (See ICLAC register)

No commonly misidentified cell lines (listed in the ICLAC register) were used in this study.

Animals and other research organisms

Policy information about studies involving animals; ARRIVE guidelines recommended for reporting animal research, and Sex and Gender in Research

Laboratory animals

The study involved domestic cattle (Bos taurus). Sexually mature, healthy female cattle (heifers) served as embryo transfer recipients. Embryo transfer procedures produced day-18 conceptuses, tissue samples, and live female offspring.

Wild animals

The study did not involve wild animals.

Reporting on sex

All experimental conceptuses and full-term offspring were female, derived from female primary bovine fetal fibroblasts (BFFs) via somatic cell nuclear transfer (SCNT). Recipient animals were adult females (Bos taurus). Details have been included in 'Ethical approval and animal use' section.

Field-collected samples

The study did not involve field-collected samples.

Ethics oversight

All animal procedures were conducted in compliance with European Union and national guidelines for animal experimentation and were approved by the local animal welfare committee (Government of Upper Bavaria, Germany). Details have been provided in the 'Ethical approval and animal use' section.

Note that full information on the approval of the study protocol must also be provided in the manuscript.

Plants

Seed stocks	n/a
Novel plant genotypes	n/a
Authentication	n/a

ChIP-seq

Data deposition

☐ Confirm that both raw and final processed data have been deposited in a public database such as GEO.

☐ Confirm that you have deposited or provided access to graph files (e.g. BED files) for the called peaks.

Data access links<br>*May remain private before publication.*	n/a
Files in database submission	n/a
Genome browser session<br>(e.g. UCSC)	n/a

Methodology

Replicates	n/a
Sequencing depth	n/a
Antibodies	n/a
Peak calling parameters	n/a
Data quality	n/a
Software	n/a
