# Supplementary material for: Pregnancy establishment in cattle without embryonic interferon tau
Source: Nat Commun. 2026 Oct 8;17:10609. doi: 10.1038/s41467-026-78446-4 (PMC13645731; doi:10.1038/s41467-026-78446-4)
Supplement: Supplementary file 2 [file 41467_2026_78446_MOESM13_ESM.pdf]

nature portfolio

Peer Review File

Pregnancy establishment in cattle without embryonic interferon tau

Corresponding Author: Professor Eckhard Wolf

This file contains all reviewer reports in order by version, followed by all author rebuttals in order by version.

Version 0:

Reviewer comments:

Reviewer #1

(Remarks to the Author)
This paper, which reports the outcome of ablating the entire functional IFNT locus on pregnancy establishment in cattle, is of great importance, since it negates the accepted paradigm for IFNT function as an essential player in pregnancy recognition in cattle and, most likely, all ruminant ungulates. Thus, IFNT may not be strictly required for pregnancy success, i.e. birth of live calves. However, it is important to note that all earlier function studies emphasized that IFNT infusion into the uterus could only prolong estrous cycle length, i.e. luteal maintenance, for a relatively short period (days rather than months) and that other agents were likely at work. This and alternative studies in sheep will spur researchers to seek other signals from the conceptus and/or alternative maternal signaling pathways that accomplish what was initially attributed to the actions of IFNT.
The authors demonstrate that transcripts for the three main expressed isoforms of boIFNT are not expressed in knock out conceptuses, that the conceptuses do not secrete boIFNT and most probably other Type I IFNT, and that the IFN signaling pathways in the uterine endometrium do not become upregulated in uterine endometrium of the pregnant cattle. All this is quite convincing, but some additional information is needed before the paper should be accepted for publication.
1. Although it seems clear that there was successful ablation of the three expressed IFNT genes in the bovine fibroblast line(s) used for donor nuclei, this reviewer believes more details should be provided about the cells, as well as the locus targeted. Although it is stated that multiple fetal fibroblast cultures were established from different fetuses, it is not clear whether a single or multiple lines, contributed as donors in SCNT. Also, what gene database was employed to map the locus? Was it assembly 3.1, from which Walker & Roberts (BMC Genomics 10, 2009) first annotated the bovine IFN type I locus, including the order of the IFNT and the various pseudogenes.
2. The authors state that 212 knockout embryos and 144 WT embryos were used in their experiments and that the two kinds developed comparably. However, the graphs from Fig. 2a show only 4 KO embryos and 5 WT embryos, with wide variance for both cleavage and blastocyst numbers. Are these data from a single or separate experiment and are they sufficient for drawing the conclusions stated? Was time to reach blastocyst measured, and could the information be provided? What was the fate of embryos that were not used in these and in the embryo transfer experiments? Were any comparable measurements made on these embryos in culturing them to blastocyst? The long-term culture experiments are also puzzling, as, to my knowledge, normal in vivo elongation and development out to day 21 has not been achieved with bovine blastocysts in vitro. Was a microscopic assessment of the embryos performed after culture? How did they look? I also note that only two KO
embryos contributed to the time course for IFNT production. Why so few when so many were available? 3. In the embryo transfer experiments, how many total recipients received embryos and what was the pregnancy success rate (say to day 30)? Finally, full term was anticipated to be February 2026, about the time I am reviewing this manuscript. Have any calves been born and could these data be included with the paper?
Minor Issues:
1. The last paragraph of the Introduction (lines 89-100) is redundant. It merely repeats what was stated in the Summary and Discussion.
2. The authors are fond of using the participle “using” repetitively and incorrectly. For example, consider the first on line 107: Candidate IFNT-KO 107 single-cell clones were screened using three PCR assays targeting distinct regions. The PCR clones did not use anything; you the authors did. The participle is “dangling”, i.e. dissociated from the user. This is one of the perils of the passive voice. It would be OK to state “we screened the clones using PCR assays”, but a simpler solution might be to insert the preposition “by” before the participle. I should add that the paper is otherwise well written, and that this error is found in a majority of modern biomedical publications, but that does not mean it should be tolerated.

Final Points:
1. I would suggest that in the revision, in some manner pay proper attribution to the work of Chris Davies and his group at Utah State University who used an analogous genetic model in sheep, namely ablation of IFNAR2, and achieved essentially a similar outcome to here, namely lack of IFNT signaling and successful pregnancy recognition. I recognize the difficulty of referencing an abstract or a funded grant containing results that have not been peer-reviewed but I'm sure the editors can help.
2. There are instances where referencing an original report might be better for the reader than a rather obscure later review. One example is the identification of boIFNT as an interferon and ultimately allowing the recombinant protein to be produced, which was achieved by expression cloning of its cDNA by Imakawa et al. (Molecular Endocrinology 3, 128-139: 1989

Reviewer #2

(Remarks to the Author)
Summary
Interferon tau (IFNT) has been hypothesized for many decades to be the pregnancy recognition signal in ruminants that is produced by the trophectoderm of the elongating conceptus. The authors here directly tested by genetic loss-of-function in bovine embryos. Bovine embryos lacking all functional IFNT gene copies were created and assessed for developmental competence and ability to establish pregnancy. The striking results found that the IFNT null embryos were developmentally competent to establish and maintain pregnancy to term. These results provide direct genetic evidence that embryonic IFNT is not required for pregnancy establishment in cattle, fundamentally challenging and revising the long-standing model of maternal recognition of pregnancy in ruminants.

Overall and Major Comments
This manuscript contains some very interesting findings of appropriate and well-designed studies. Careful review generated several major and on specific comment.

Major Comments:
(1) Figure 2: Can the authors provide more details on how the bovine embryos were cultured for 11 to 19 days and assessed for IFNT? The data should be expressed as total amount of IFNT in the culture media normalized for embryo number.
(2) Figure 3: Did the authors sequence day 18 WT and IFNT KO conceptuses? This would be interesting to see if there was some compensation (or lack) in other genes due to the absence of IFNT.
(3) Discussion: The discussion lacks information on how this extraordinary finding impacts our understanding of MRP. What do the authors propose is the signal for MRP in cattle? Could it be prostaglandins produced by the elongating bovine conceptus?
(4) Discussion: Brooks and Spencer (Biol Reprod 2015; 92:47) found that IFNT has a biological role in sheep conceptus elongation based on a morpholino loss-of-function approach. Is it possible that sheep and cattle have basic differences in the biology of IFNT actions? The authors should discuss this finding in relation to their results in cattle.

Specific Comments
Line Comment
70 The data in sheep does not indicate that IFNT suppresses expression of estrogen receptor alpha (ESR1) or oxytocin receptors (OXTR). More precisely, IFNT inhibits the upregulation of ESR1 and OXTR in the endometrial luminal epithelium that normally occurs in cyclic ewes. This work was done by Fuller W. Bazer and Thomas E. Spencer at Texas A&M University.

Reviewer #3

(Remarks to the Author)
This is a potentially paradigm-shifting set of experiments that call into question the dogma that conceptus interferons are responsible for maintenance of pregnancy in ruminants. The experiments are very well done. It is clear from the results that the knockouts eliminated interferon-tau, that embryos can develop in vitro normally, and can elongate in vivo normally. An additional experiment in which knockout embryos were transferred and allowed to develop in utero up to 75 days of gestation is more problematic. It is clear that embryos could block luteolysis and continue normal development up to that time. Experiments are well designed and the paper elegantly written.

There is one flaw in the design of this last experiment, however, that allows for two alternative interpretations. The flaw is that two knockout embryos were transferred into each cow. The fact that pregnancy continued to day 75 is interpreted by the authors as meaning IFNT is not a necessary maternal recognition of pregnancy signal. There is a second explanation, however, that also fits the observed data. It is possible that IFNT is the primary maternal recognition of pregnancy signal in cattle and that secondary signals which may facilitate maternal recognition of pregnancy (for example prostaglandin E2 or pregnancy associated proteins), are not able by themselves to maintain the corpus luteum when a single embryo is present in the reproductive tract (which is the normal situation in cattle). However, these secondary signals are able to maintain the corpus luteum when abnormally high amounts are secreted into the uterine lumen due to the presence of two embryos in the uterus.

The author would be well served to repeat their last experiment except where only one embryo is transferred into each recipient. If knockout embryos develop to day 50 or 75, they will have conclusvely shown that IFNT is dispensable. As the

research stands now, reasonable doubt remains.

Version 1:

Reviewer comments:

Reviewer #1

(Remarks to the Author)
The manuscript is much improved, and the authors addressed all my comments thoroughly and completely. It nicely complements the work of Davies et al on a sheep model in which IFNAR2 has been deleted.

Reviewer #2

(Remarks to the Author)
The authors have adequately and quite expertly revised the initial manuscript to address all concerns and questions raised by this reviewer. Very well done experiments and interesting novel outcome.

Reviewer #3

(Remarks to the Author)
The additional experiment in which a single embryo was transferred cinches the case that IFNT is not required for pregnancy establishment. Good job. This is indeed a paradigm-shifting paper.

Open Access This Peer Review File is licensed under a Creative Commons Attribution 4.0 International License, which permits use, sharing, adaptation, distribution and reproduction in any medium or format, as long as you give appropriate credit to the original author(s) and the source, provide a link to the Creative Commons license, and indicate if changes were made.
In cases where reviewers are anonymous, credit should be given to 'Anonymous Referee' and the source.
The images or other third party material in this Peer Review File are included in the article's Creative Commons license, unless indicated otherwise in a credit line to the material. If material is not included in the article's Creative Commons license and your intended use is not permitted by statutory regulation or exceeds the permitted use, you will need to obtain permission directly from the copyright holder.
To view a copy of this license, visit https://creativecommons.org/licenses/by/4.0/

POINT-BY-POINT RESPONSE TO REVIEWERS' COMMENTS

Reviewer #1:

This paper, which reports the outcome of ablating the entire functional IFNT locus on pregnancy establishment in cattle, is of great importance, since it negates the accepted paradigm for IFNT function as an essential player in pregnancy recognition in cattle and, most likely, all ruminant ungulates. Thus, IFNT may not be strictly required for pregnancy success, i.e. birth of live calves. However, it is important to note that all earlier function studies emphasized that IFNT infusion into the uterus could only prolong estrous cycle length, i.e. luteal maintenance, for a relatively short period (days rather than months) and that other agents were likely at work. This and alternative studies in sheep will spur researchers to seek other signals from the conceptus and/or alternative maternal signaling pathways that accomplish what was initially attributed to the actions of IFNT.

The authors demonstrate that transcripts for the three main expressed isoforms of boIFNT are not expressed in knock out conceptuses, that the conceptuses do not secrete boIFNT and most probably other Type I IFNT, and that the IFN signaling pathways in the uterine endometrium do not become upregulated in uterine endometrium of the pregnant cattle. All this is quite convincing, but some additional information is needed before the paper should be accepted for publication.

We thank the reviewer for the positive assessment of our work and for the insightful and constructive comments, which have significantly improved the manuscript. We have carefully addressed all points raised, with the corresponding changes highlighted in yellow in the revised version. Below is our point-by-point response.

1. Although it seems clear that there was successful ablation of the three expressed IFNT genes in the bovine fibroblast line(s) used for donor nuclei, this reviewer believes more details should be provided about the cells, as well as the locus targeted. Although it is stated that multiple fetal fibroblast cultures were established from different fetuses, it is not clear whether a single or multiple lines, contributed as donors in SCNT. Also, what gene database was employed to map the locus? Was it assembly 3.1, from which Walker & Roberts (BMC Genomics 10, 2009) first annotated the bovine IFN type I locus, including the order of the IFNT and the various pseudogenes.

We thank the reviewer for this important comment.

We would like to clarify that a single bovine fetal fibroblast (BFF) line was used to generate the two IFNT-KO single-cell clones (SCC-22 and SCC-34).

We have now expanded the Methods section for greater clarity. Lines 389-398 contain the following text about the cells and the target locus:

'Primary bovine fetal fibroblast cells (BFFs), internally designated as BFF-091019, were established from an early-gestation female fetus obtained from the slaughterhouse following standard protocol. Cells were maintained under standard culture conditions at 37°C with controlled humidity and 5% CO_2 in fibroblast growth medium supplemented with 15% fetal calf serum (FCS), non-essential amino acids, and HEPES buffer. The single-guide RNA (sgRNA) design and genomic locus annotation were based on the latest ARS-UCD2.0 bovine genome assembly (NCBI RefSeq assembly GCF_002263795.3; GenBank assembly GCA_002263795.4) submitted by

Rosen et al [52,53]. The targeted region included all six alleles of the three functional *IFNT* genes: *IFN-tau-c1* (Gene ID: 528153), *IFNT2* (Gene ID: 317698), and *IFNT3* (Gene ID: 100313956). '

Moreover, lines 418-427 contain additional details about the donor single-cell clones:
'Clones lacking amplification across all three *IFNT*-specific assays, indicative of complete deletion of all *IFNT* copies, while retaining *GAPDH* product, were designated as complete knockouts. Starting with the single parental BFF-091019 population, multiple single-cell clones were generated and screened for complete *IFNT*-KO. Following comprehensive molecular validation, two independent *IFNT*-KO single-cell clones (SCC-22 and SCC-34) were selected for all subsequent in vitro functional assays and in vivo embryo transfer experiments on the basis of their confirmed complete deletion of all functional *IFNT* alleles and superior growth characteristics, including morphology and proliferation rate. Unedited parental BFF-091019 cells served as WT controls throughout genotyping and SCNT procedures.'

2. The authors state that 212 knockout embryos and 144 WT embryos were used in their experiments and that the two kinds developed comparably. However, the graphs from Fig. 2a show only 4 KO embryos and 5 WT embryos, with wide variance for both cleavage and blastocyst numbers. Are these data from a single or separate experiment and are they sufficient for drawing the conclusions stated? Was time to reach blastocyst measured, and could the information be provided? What was the fate of embryos that were not used in these and in the embryo transfer experiments? Were any comparable measurements made on these embryos in culturing them to blastocyst?

The authors would like to clarify that multiple SCNT sessions using KO and WT cells were performed; each data point in Figure 2a represents the percentage of embryos that progressed to a specific stage in an independent SCNT session, and comparable measurements were made between the KO and WT groups.

To address all the raised questions, the following details and additional data have been added to the manuscript in the heading 'Normal in vitro development of IFNT-null embryos', in lines 125-145:

'Each data point in Fig. 2a represents the percentage of embryos that progressed to a specific developmental stage (fusion, cleavage, or blastocyst) within one independent SCNT session. In total, five independent SCNT sessions were performed with *IFNT*-KO donor cells and five sessions with WT donor cells. Detailed embryo numbers, fusion rates, cleavage rates, and blastocyst formation rates for each session are provided in **Extended Data Table 1**. In sum, these experiments yielded a total of 212 reconstructed *IFNT*-KO embryos and 144 WT embryos. Fusion, cleavage, and blastocyst formation rates were comparable between groups, indicating that *IFNT*-KO does not affect in vitro blastocyst development (**Fig. 2a**). Microscopic evaluation of the blastocysts was performed daily; early blastocysts were first observed on day 5, expanded blastocysts on day 6, and hatching or fully hatched blastocysts on day 7-8 (**Extended Data Fig. 1a)**, with no difference in developmental timing between the *IFNT*-KO and WT groups. High-quality hatched blastocysts were selected on day 7 for transfer to synchronized recipients. Some of the remaining blastocysts were utilized for long-term in vitro culture experiments, with each embryo cultured individually in an isolated microdrop. It is important to note that the long-term in vitro cultures were performed solely to assess IFNT secretion into the medium and were not intended

to evaluate the effect of IFNT loss on post-blastocyst developmental potential. While a bovine embryo cannot develop normally under the currently employed in vitro conditions, the trophoblast cells proliferate and secrete IFNT [31]. Microscopic examination of embryos after extended culture confirmed the expected morphology (**Extended Data Fig. 1b)**.'

The figure legend (2a) has been updated as:

'a, Developmental competence of *IFNT*-KO and WT embryos generated by SCNT. In total, five independent SCNT sessions were performed with *IFNT*-KO donor cells and five sessions with WT donor cells, yielding a total of 212 reconstructed *IFNT*-KO embryos and 144 WT embryos. Each data point represents the percentage of embryos that progressed to a specific developmental stage (fusion, cleavage, or blastocyst) in a single independent SCNT session. Detailed embryo numbers and developmental outcomes for each session are provided in Extended Data Table 1. Fusion rate, cleavage rate, and blastocyst formation rate were comparable between groups.'

The long-term culture experiments are also puzzling, as, to my knowledge, normal in vivo elongation and development out to day 21 has not been achieved with bovine blastocysts in vitro. Was a microscopic assessment of the embryos performed after culture? How did they look?

We thank the reviewer for this observation. We fully agree that extended in vitro culture of bovine blastocysts does not recapitulate normal in vivo elongation and development to day 21. The primary purpose of the long-term culture experiments was to determine whether *IFNT*-KO embryos secrete detectable IFNT protein into the medium compared with WT embryos, rather than to assess post-blastocyst developmental competence. Microscopic assessment of embryos after long-term culture confirmed that they showed the expected signs of degeneration under standard culture conditions. To address this important point for readers, we have added the following text in the Results section in the lines '138-145':

'Some of the remaining blastocysts were utilized for long-term in vitro culture experiments, with each embryo cultured individually in an isolated microdrop. It is important to note that the long-term in vitro cultures were performed solely to assess IFNT secretion into the medium and were not intended to evaluate the effect of IFNT loss on post-blastocyst developmental potential. While a bovine embryo cannot develop normally under the currently employed in vitro conditions, the trophoblast cells proliferate and secrete IFNT [31]. Microscopic examination of embryos after extended culture confirmed the expected morphology (**Extended Data Fig. 1b)**.'

I also note that only two KO embryos contributed to the time course for IFNT production. Why so few when so many were available?

The complete knockout of IFNT was first confirmed at the genomic level by multiple PCR assays (Fig. 1c). Functional absence of IFNT was then robustly demonstrated using a viral cytopathic effect (CPE) inhibition bioassay on media from a large number of embryos (Fig. 2b). The Western blot and LC-MS/MS analyses (Fig. 2c,d) served as additional orthogonal confirmatory methods. For these specific assays, an independent SCNT session was performed using the same two validated *IFNT*-KO clones. Because individual embryos exhibit variable survival during extended in vitro culture, conditioned media were collected longitudinally from the longest-surviving embryos. In this session, two *IFNT*-KO embryos remained viable through day 21, which is why only these two were included in the full time-course Western blot and mass spectrometry analyses.

The CPE bioassay included substantially more embryos and confirmed the complete absence of antiviral activity across all KO samples.

To highlight this, the following additional note has been added to the legends of Figure 2:

'**Note:** For Western blot and LC-MS/MS analyses (c, d), conditioned media were collected longitudinally from individually cultured embryos in an independent SCNT session. Only two SCNT-derived *IFNT*-KO embryos survived to day 21; data from these longest-surviving embryos are shown. The antiviral bioassay (b) included substantially more embryos and confirmed the complete absence of IFNT activity across all KO samples. Because embryos were cultured individually, all IFNT values in panels b-d represent secretion per embryo.'

3. In the embryo transfer experiments, how many total recipients received embryos, and what was the pregnancy success rate (say to day 30)? Finally, the full term was anticipated to be February 2026, about the time I am reviewing this manuscript. Have any calves been born, and could these data be included with the paper?

To investigate pregnancy survival beyond day 18, we performed two rounds of embryo transfers. In the first round, we used 10 recipients; in the second, 4.

We are happy to report that two healthy female calves were born in February, 2026.

To address the points raised by the reviewer, we have added supplementary figures and tables that provide detailed outcomes for each animal and the overall outcome.

Lines 197-205:

'To this end, an independent series of embryo transfers was performed with SCNT-derived *IFNT*-KO blastocysts. Ten estrus-synchronized recipient heifers received either two (n = 8) or three (n = 2) *IFNT*-KO blastocysts per animal. Estrus behavior was monitored via automated neck-collar accelerometers and processed by the Lely Horizon management platform (Lely, Maassluis, Netherlands), generating time-series activity profiles to map estrus-associated behavioral dynamics (**Extended Data Fig. 3**). Two recipients (67914 and 67929) that received three embryos each returned to estrus within 20-21 days. In contrast, the remaining eight recipients (80%) exhibited prolonged luteal phases (**Extended Data Table 5**).'

Lines 227-238:

'To directly address whether a single *IFNT*-null conceptus is sufficient to establish and sustain pregnancy, we performed a subsequent round of transfers in which 4 synchronized recipients each received a single *IFNT*-KO blastocyst. Transrectal ultrasonography performed on day 36 of gestation confirmed a successful pregnancy in one recipient, which was subsequently confirmed on day 46 (**Extended Data Fig. 6**; **Extended Data Video 2**; **Extended Data Table 7**), with the pregnancy remaining ongoing. This result definitively demonstrates that even a single *IFNT*-null conceptus is fully capable of preventing luteolysis and sustaining pregnancy, thereby ruling out the possibility that successful outcomes in the transfers with two blastocysts could be due to a dose-dependent effect of redundant secondary signals. Across all experimental cohorts, the overall ultrasound-confirmed pregnancy rate was 4/14 (28.6%), representing a remarkably high developmental efficiency for an SCNT-derived gene-knockout model.'

The following information about the live-born calves has been added in lines 223-226:

'The third twin pregnancy progressed successfully to term, culminating in the birth of two healthy female calves on February 26, 2026. Both calves were confirmed to be *IFNT*-null by genotyping (**Fig. 4i, j**) and showed physiological parameters within normal ranges (**Extended Data Table 6**).'

Minor Issues:

1. The last paragraph of the Introduction (lines 89-100) is redundant. It merely repeats what was stated in the Summary and Discussion.

We have replaced this paragraph with a non-redundant shortened version (lines 96-100).

2. The authors are fond of using the participle "using" repetitively and incorrectly. For example, consider the first on line 107: Candidate IFNT-KO 107 single-cell clones were screened using three PCR assays targeting distinct regions. The PCR clones did not use anything; you, the authors, did. The participle is "dangling", i.e. dissociated from the user. This is one of the perils of the passive voice. It would be OK to state "we screened the clones using PCR assays", but a simpler solution might be to insert the preposition "by" before the participle. I should add that the paper is otherwise well written, and that this error is found in a majority of modern biomedical publications, but that does not mean it should be tolerated.

We have carefully revised the manuscript to address this comment.

Final Points:

I would suggest that in the revision, in some manner pay proper attribution to the work of Chris Davies and his group at Utah State University who used an analogous genetic model in sheep, namely ablation of IFNAR2, and achieved essentially a similar outcome to here, namely lack of IFNT signaling and successful pregnancy recognition. I recognize the difficulty of referencing an abstract or a funded grant containing results that have not been peer-reviewed but I'm sure the editors can help.

We thank the reviewer for this valuable suggestion and for highlighting the important work by Chris Davies and colleagues. We have now cited their work, including the recent 2026 study in the Introduction (lines 91-95) and provided a detailed discussion of their IFNAR2 knockout sheep model in the Discussion section (lines 265-280), comparing their approach with our ligand-level knockout in cattle.

There are instances where referencing an original report might be better for the reader than a rather obscure later review. One example is the identification of boIFNT as an interferon and ultimately allowing the recombinant protein to be produced, which was achieved by expression cloning of its cDNA by Imakawa et al. (Molecular Endocrinology 3, 128-139: 1989.

We completely agree that citing the original discoveries enhances the manuscript's scholarly accuracy. We have cited the foundational reports, including the original cloning of ovine IFNT (Imakawa et al., Nature, 1987), the subsequent cloning of bovine IFNT (Imakawa et al., Mol. Endocrinol., 1989), and the mechanistic papers demonstrating the silencing of epithelial ESR1 and OXTR (Spencer and Bazer, J. Reprod. Fertil., 1995; Spencer and Bazer, Endocrinology, 1996).

Reviewer #2 (Remarks to the Author):

Summary

Interferon tau (IFNT) has been hypothesized for many decades to be the pregnancy recognition signal in ruminants that is produced by the trophectoderm of the elongating conceptus. The authors here directly tested by genetic loss-of-function in bovine embryos. Bovine embryos lacking all functional IFNT gene copies were created and assessed for developmental competence and ability to establish pregnancy. The striking results found that the IFNT null embryos were developmentally competent to establish and maintain pregnancy to term. These results provide direct genetic evidence that embryonic IFNT is not required for pregnancy establishment in cattle, fundamentally challenging and revising the long-standing model of maternal recognition of pregnancy in ruminants.

We thank the reviewer for the positive evaluation and the constructive suggestions that have strengthened the manuscript. We have carefully addressed all comments raised, with the corresponding changes highlighted in yellow in the revised version. Below is our point-by-point response.

Overall and Major Comments

This manuscript contains some very interesting findings of appropriate and well-designed studies. Careful review generated several major and on specific comment.

Major Comments:

Figure 2: Can the authors provide more details on how the bovine embryos were cultured for 11 to 19 days and assessed for IFNT? The data should be expressed as the total amount of IFNT in the culture media normalized for embryo number.

We would like to clarify that the embryos were cultured individually in separate drops, and the presented IFNT concentrations in the conditioned medium directly represent secretion per embryo.

For further clarity and to address the reviewer's comments, the following text has been added to the Methods section, lines 473-481:

'Long-term culture of bovine embryos

To analyze the release of IFNT by the embryos, long-term in vitro culture of embryos was performed following our previously published protocol [55,56]. Briefly, day-8 SCNT or IVF blastocysts were individually placed into 60-µL drops of Ham's F12 medium supplemented with 10^{-4} M 2-mercaptoethanol (Merck, Darmstadt, Germany) and 15% FCS. Culture was carried out at 37°C in humidified air with 10% CO_2. On Days 11, 15, 19, and 21, 30 µL of medium from each drop was collected for IFNT quantification, and the same volume of fresh culture medium was added to replace it. Samples were stored individually at -80°C until further analysis. Because each embryo was cultured individually, the measured IFNT concentrations in the conditioned medium directly represent secretion per embryo.'

The following text has been added to the results in lines 138-139:

‘Some of the remaining blastocysts were utilized for long-term in vitro culture experiments, with each embryo cultured individually in an isolated microdrop.’

(2) Figure 3: Did the authors sequence day 18 WT and IFNT KO conceptuses? This would be interesting to see whether there was any compensation (or lack thereof) in other genes due to the absence of IFNT.

We thank the reviewer for this important suggestion. We would like to clarify that we had already performed quantitative proteomic analysis of the day-18 conceptuses, as shown in Figure 3e and Extended Dataset 1.

To further address this point, we have now conducted RNA sequencing and targeted prostaglandin analysis on the same conceptus samples.

The methods used for these analyses are provided in lines 566-605.

These additional datasets reinforce our original findings: global transcriptomic and proteomic profiles were highly similar between IFNT-KO and wild-type conceptuses, with no evidence of compensatory upregulation of alternative interferons or related pathways. Similarly, prostaglandin levels (including PGE_2) and the expression of key prostaglandin-synthesis enzymes showed no significant differences between groups. Collectively, these multi-omics data argue against a simple compensatory mechanism within the conceptus and instead support the existence of distinct, autonomous, and IFNT-independent pathways capable of sustaining pregnancy.

The following text has been included in the results section in lines 174-184:

‘Quantitative proteomic analysis of conceptus tissues revealed no global differences in protein abundance between *IFNT*-KO and WT groups (**Fig. 3e**; **Extended Dataset 1**); notably, no IFNT-derived peptides were detected in any *IFNT*-KO samples (**Extended Data Table 3**). Complementary transcriptomic profiling of the same conceptuses likewise showed no significant differences in gene expression between groups (**Extended Dataset 2**). In addition, targeted analysis of prostaglandins in conceptus tissues revealed no differences in the levels of PGD_2, PGE_1, PGE_2, $PGF_1α$, or $PGF_2α$ (**Extended Data Fig. 2**). Similarly, transcript and protein levels of the prostaglandin E synthases (PTGES, PTGES2, and PTGES3) were comparable between the two genotypes (**Extended Data Table 3**), indicating that the absence of IFNT does not trigger compensatory upregulation of prostaglandin synthesis or any other compensatory responses within the conceptus.’

The following text has been included in the discussion in lines 281-289:

‘Surprisingly, quantitative global proteomics and transcriptomics of day-18 *IFNT*-KO and WT conceptuses revealed highly similar molecular profiles, with no evidence of upregulated alternative interferons or aberrant induction of collateral signaling pathways. These congruent molecular profiles underscore the high on-target efficiency and specificity of the sgRNAs utilized for *IFNT* ablation, confirming the lack of biologically relevant off-target effects. Moreover, the lack of intra-conceptus adaptation argues against a simple compensatory mechanism. Instead, it suggests the existence of a distinct, autonomous, and IFNT-independent pathway that operates natively in both KO and WT conceptuses to drive maternal recognition of pregnancy, support conceptus elongation, and protect the corpus luteum.’

(3) Discussion: The discussion lacks information on how this extraordinary finding impacts our understanding of MRP. What do the authors propose is the signal for MRP in cattle? Could it be prostaglandins produced by the elongating bovine conceptus?

We thank the author for this very insightful comment. To address this, we performed targeted analysis of prostaglandins in conceptus tissues. We have described our findings in the results (lines 178-182):

'In addition, targeted analysis of prostaglandins in conceptus tissues revealed no differences in the levels of PGD_2, PGE_1, PGE_2, $PGF_1\alpha$, or $PGF_2\alpha$ (**Extended Data Fig. 2**). Similarly, transcript and protein levels of the prostaglandin E synthases (PTGES, PTGES2, and PTGES3) were comparable between the two genotypes (**Extended Data Table 3**)'.

We have also included a detailed discussion on PGE_2 being a possible alternate MRP signal and have added the following text in the discussion in lines 290-309:

'A compelling mediator of this alternative embryo-maternal dialogue is an embryo-driven prostaglandin signaling cascade. Historically, intrauterine infusion of exogenous prostaglandin E_2 (PGE_2) alone has proven sufficient to extend luteal lifespan and mimic the presence of a conceptus in ewes [33], heifers [34], and gilts [35]. Mechanistically, the uterine and conceptus-derived PGE_2 pool is actively transported via a local countercurrent mechanism through the utero-ovarian plexus (UOP) directly to the ovary [36]. Upon reaching the ovary, this influx acts as a critical signal that shifts the intraluteal PGE_2 to $PGF_2\alpha$ ratio away from luteolysis ($PGF_2\alpha$-dominant) and toward luteal preservation (PGE_2-dominant) [36].

Our targeted metabolomic and molecular analyses directly substantiate this hypothesis. PGE_2 showed robust accumulation in day-18 peri-implantation conceptuses, with levels statistically indistinguishable between the *IFNT*-KO and WT cohorts. Concordantly, the primary terminal enzymes critical for PGE_2 biosynthesis (PTGES, PTGES2, and PTGES3) were robustly and equivalently expressed across both cohorts. This expression signature aligns with conserved mammalian strategies, such as those observed in suids, where the early conceptus strongly upregulates key prostaglandin synthesis enzymes, including PTGES, to autonomously subvert maternal luteolytic signaling [37]. We therefore speculate that the elongating bovine conceptus utilizes this conserved prostaglandin signaling network to drive a localized luteoprotective environment, characterized by an elevated PGE_2-to-$PGF_2\alpha$ ratio. Nonetheless, comprehensive temporal profiling of embryonic PGE_2 dynamics and formal genetic loss-of-function investigations remain essential to definitively establish its obligatory role during MRP in cattle.'

To highlight the long-reaching impact of this study, we have added the following closing paragraph in lines 341-352:

'From a translational perspective, our findings prompt a fundamental paradigm shift in how we approach and treat infertility in ruminants. For decades, diagnostic strategies and therapeutic interventions aimed at mitigating early pregnancy loss have focused almost exclusively on monitoring or supplementing IFNT signaling. Our results demonstrate that this historical focus targets a non-obligatory, parallel pathway rather than the real driver of pregnancy recognition. By demonstrating that early gestation can be established and maintained beyond key developmental milestones in the complete absence of a maternal interferon response, these data reveal that the

ruminant embryo relies on a distinct, autonomous, and self-sufficient signaling axis to override the maternal luteolytic clock. Consequently, the IFNT-deficient model developed in this study provides a powerful platform for identifying and validating alternative maternal recognition pathways in domestic livestock and for developing novel strategies to improve reproductive efficiency.'

(4) Discussion: Brooks and Spencer (Biol Reprod 2015; 92:47) found that IFNT has a biological role in sheep conceptus elongation based on a morpholino loss-of-function approach. Is it possible that sheep and cattle have basic differences in the biology of IFNT actions? The authors should discuss this finding in relation to their results in cattle.

To address these comments and compare our results with the data presented by Brooks and Spencer (2015) and Davies et al (2022), we have included the following detailed discussion in lines 256-280:

'*IFNT*-KO conceptuses recovered at the peri-implantation stage (day 18) were morphologically indistinguishable from WT controls and exhibited comparable elongation. This finding contrasts with a previous report in sheep, where a morpholino antisense oligonucleotide targeting *IFNT* resulted in compromised conceptus elongation, suggesting an active autocrine role for IFNT during early development [6]. While species-specific differences remain a possibility, this phenotypic divergence is more likely explained by the profound technical distinctions between transient translational knockdowns and stable genetic knockouts. In mammalian embryology, morpholino-mediated knockdowns frequently introduce confounding off-target artifacts or cellular stress that do not emerge in clean genetic models [32].

Our findings regarding the dispensability of autocrine IFNT signaling for conceptus elongation are strongly corroborated by a genetic model in sheep, in which embryos with a complete genomic knockout of *IFNAR2* developed normally and yielded viable rams [29]. Because IFNAR2 is thought to be mandatory for IFNT signaling, its ablation renders the developing embryo functionally blind to any autocrine effects of its own conceptus-derived IFNT.

Following the publication of our findings, Davies et al. (2026) reported that *IFNAR2*-KO ewes are fertile and can establish successful pregnancies through natural breeding, giving birth to viable lambs [31]. Notably, the parents and hence fetuses in these pregnancies had intact *IFNT*. Thus, while this study further challenges the absolute requirement for IFNT-IFNAR signaling, it does not directly address the paracrine action of conceptus-derived IFNT on the maternal endometrium during MRP. An intriguing possibility could be the existence of an alternative IFNT receptor. By contrast, our genetic loss-of-function model in cattle provides the first direct, ligand-level confirmation that the IFNT-IFNAR signaling axis is entirely dispensable for pregnancy establishment and maintenance in ruminants. Nevertheless, generating a complementary ovine model with complete genetic ablation of *IFNT* remains essential to establish a definitive cross-species conclusion.'

Specific Comments

Line Comment

70 The data in sheep does not indicate that IFNT suppresses expression of estrogen receptor alpha (ESR1) or oxytocin receptors (OXTR). More precisely, IFNT inhibits the upregulation of

ESR1 and OXTR in the endometrial luminal epithelium that normally occurs in cyclic ewes. This work was done by Fuller W. Bazer and Thomas E. Spencer at Texas A&M University.

We have revised the text as follows (lines 73-75):

‘Its antiluteolytic effect is thought to be mediated by the interferon regulatory factor 2 (IRF2)-dependent inhibition of estrogen receptor alpha (*ESR1*) and oxytocin receptor (*OXTR*) upregulation within the endometrial luminal epithelium [19,20].’

Reviewer #3 (Remarks to the Author):

This is a potentially paradigm-shifting set of experiments that call into question the dogma that conceptus interferons are responsible for maintenance of pregnancy in ruminants. The experiments are very well done. It is clear from the results that the knockouts eliminated interferon-tau, that embryos can develop in vitro normally, and can elongate in vivo normally. An additional experiment in which knockout embryos were transferred and allowed to develop in utero up to 75 days of gestation is more problematic. It is clear that embryos could block luteolysis and continue normal development up to that time. Experiments are well designed and the paper elegantly written.

There is one flaw in the design of this last experiment, however, that allows for two alternative interpretations. The flaw is that two knockout embryos were transferred into each cow. The fact that pregnancy continued to day 75 is interpreted by the authors as meaning IFNT is not a necessary maternal recognition of pregnancy signal. There is a second explanation, however, that also fits the observed data. It is possible that IFNT is the primary maternal recognition of pregnancy signal in cattle and that secondary signals which may facilitate maternal recognition of pregnancy (for example prostaglandin E2 or pregnancy associated proteins), are not able by themselves to maintain the corpus luteum when a single embryo is present in the reproductive tract (which is the normal situation in cattle). However, these secondary signals are able to maintain the corpus luteum when abnormally high amounts are secreted into the uterine lumen due to the presence of two embryos in the uterus.

The author would be well served to repeat their last experiment, except where only one embryo is transferred into each recipient. If knockout embryos develop to day 50 or 75, they will have conclusively shown that IFNT is dispensable. As the research stands now, reasonable doubt remains.

We thank the reviewer for their positive comments and insightful perspective on the twin pregnancies. We have carefully addressed the raised concern through an additional set of experiments, with the corresponding changes highlighted in yellow in the revised version. Below is our response.

We would like to highlight that transferring more than one embryo per recipient is standard practice in ruminant SCNT programs, which are employed to mitigate the lower developmental competence and higher post-implantation attrition rates typically observed in cloned embryos compared to their in vitro fertilized counterparts. Moreover, the aim of this study was to determine whether a pregnancy can be initiated and maintained in the absence of the IFNT signal, which we successfully achieved.

However, we acknowledge that the initial use of multiple embryos per recipient introduced a theoretical possibility of a dose-dependent effect from redundant conceptus-derived signals. This prompted us to perform additional critical experiments transferring a single *IFNT*-KO blastocyst per animal (n=4). We can confirm that a single-embryo transfer successfully established a viable singleton pregnancy that remains healthy and ongoing beyond day 60 of gestation. This critical milestone definitively demonstrates that a single *IFNT*-null conceptus is fully sufficient to prevent

luteolysis and support extended development, effectively ruling out a simple numerical dose effect of compensatory signals.

These additional experiments and findings have been included in:

The methods section (lines 562-565):

‘An additional round of transfers was performed with four synchronized recipients, each receiving a single *IFNT*-KO blastocyst. One recipient successfully established a viable singleton pregnancy that was confirmed by ultrasonography on days 36 and 46 and remains ongoing.’

The results section (lines 227-238):

‘To directly address whether a single *IFNT*-null conceptus is sufficient to establish and sustain pregnancy, we performed a subsequent round of transfers in which 4 synchronized recipients each received a single *IFNT*-KO blastocyst. Transrectal ultrasonography performed on day 36 of gestation confirmed a successful pregnancy in one recipient, which was subsequently confirmed on day 46 (**Extended Data Fig. 6**; **Extended Data Video 2**; **Extended Data Table 7**), with the pregnancy remaining ongoing. This result definitively demonstrates that even a single *IFNT*-null conceptus is fully capable of preventing luteolysis and sustaining pregnancy, thereby ruling out the possibility that successful outcomes in the transfers with two blastocysts could be due to a dose-dependent effect of redundant secondary signals. Across all experimental cohorts, the overall ultrasound-confirmed pregnancy rate was 4/14 (28.6%), representing a remarkably high developmental efficiency for an SCNT-derived gene-knockout model.’

And the discussion (lines 325-332):

‘Although our initial embryo transfers utilized two blastocysts per recipient, one of which culminated in the birth of two live, clinically normal calves, we performed an additional round of single-embryo transfers to exclude any potential dose effect from multiple conceptuses. Transfer of a single *IFNT*-KO blastocyst successfully established a viable singleton pregnancy that continues to demonstrate normal gestational progression. Together, these findings definitively demonstrate that a single *IFNT*-null conceptus is fully sufficient to prevent luteolysis and support development, thereby ruling out a simple numerical dose effect of compensatory signals.’
