# Supplementary material for: Pregnancy establishment in cattle without embryonic interferon tau
Source: Nat Commun. 2026 Oct 8;17:10609. doi: 10.1038/s41467-026-78446-4 (PMC13645731; doi:10.1038/s41467-026-78446-4)
Supplement: Supplementary file 3 [file 41467_2026_78446_MOESM1_ESM.pdf]

Pregnancy establishment in cattle without embryonic interferon tau

Supplementary Information

Asghar Ali[1,2,*], Valeri Zakhartchenko[1,2], Tuna Güngör[1,2], Stefan Nüske[3], Jan B. Stöckl[4], Sarah Hecken[4], Andreas Hauser[4], Stefan Krebs[4], Sven Reese[5], Marcel Suchowski[6], Robert Friedl[7], Sebastian Schwanitz[3], Armin M. Scholz[3], Andreas Blutke[8], Martina Protschka[9], Mathias Büttner[9], Thomas Fröhlich[4], Horst-Dieter Reichenbach[10], Eckhard Wolf[1,2,4,*]

[1]Chair for Molecular Animal Breeding and Biotechnology, Gene Center and Department of Veterinary Sciences, LMU Munich, 81377 Munich, Germany

[2]Center for Innovative Medical Models (CiMM), LMU Munich, 85764 Oberschleißheim, Germany

[3]Livestock Center, Faculty of Veterinary Medicine, LMU Munich, 85764 Oberschleißheim, Germany

[4]Laboratory for Functional Genome Analysis (LAFUGA), Gene Center, LMU Munich, 81377 Munich, Germany

[5]Chair of Anatomy, Histology and Embryology, Department of Veterinary Sciences, LMU Munich, 80539 Munich, Germany

[6]Bavarian Health and Food Safety Authority (LGL), 85764 Oberschleißheim, Germany

[7]Vion Waldkraiburg GmbH, 84478 Waldkraiburg, Germany

[8]Institute of Veterinary Pathology, Center for Clinical Veterinary Medicine, LMU Munich, 80539 Munich, Germany

[9]Institute of Immunology, Faculty of Veterinary Medicine, University of Leipzig, 04103 Leipzig, Germany

[10]Institute for Animal Breeding, Bavarian State Research Center for Agriculture (LfL), 85586 Grub, Germany

*Corresponding authors:

Asghar Ali (asghar.ali@lmu.de)

Eckhard Wolf (ewolf@lmu.de)

Supplementary Figures 1-6

Supplementary Tables 1-4

Source Data for Supplementary Figure 2

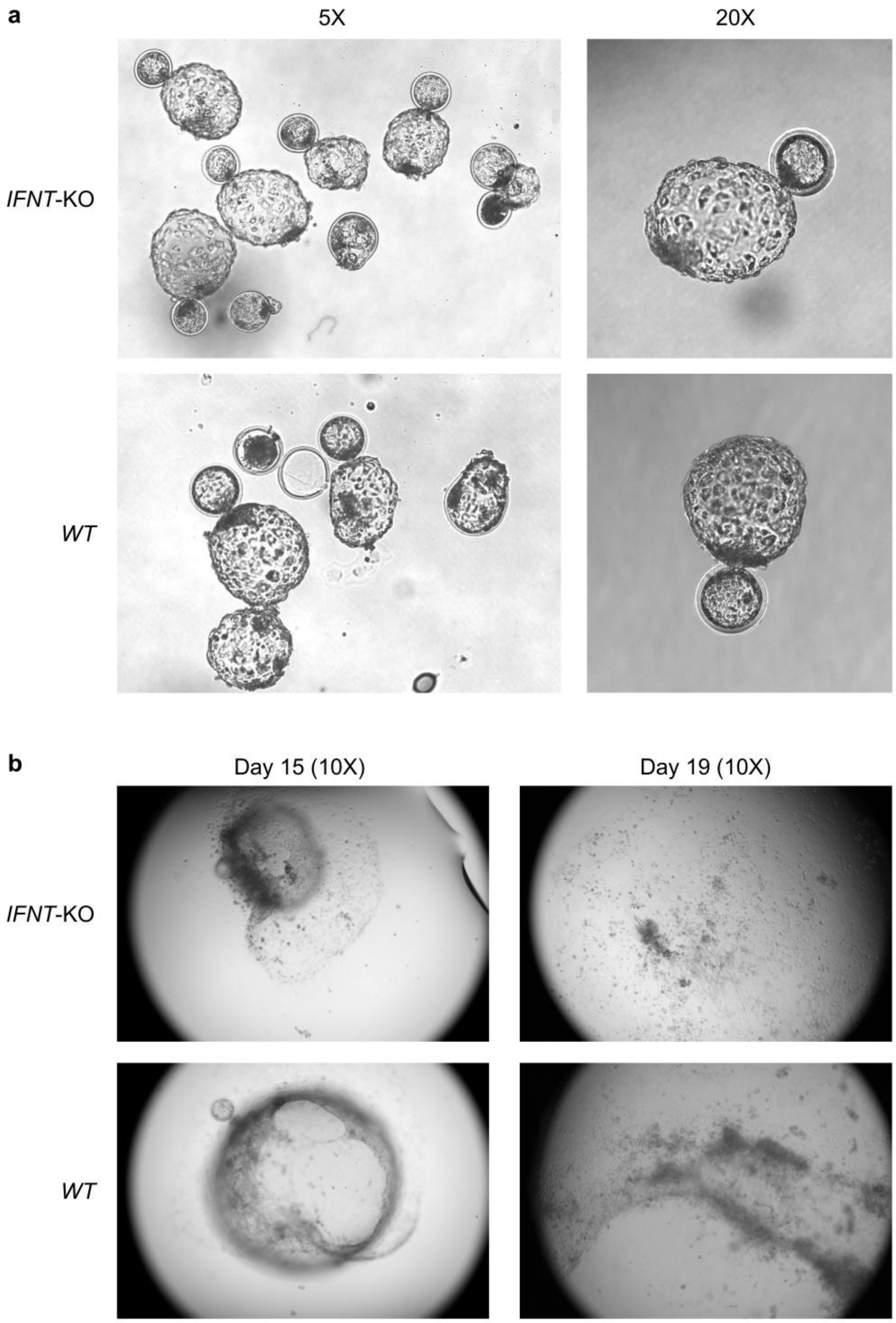

Supplementary Figure 1 | Morphology of *IFNT*-KO and wild-type (WT) embryos during in vitro culture. a, Representative bright-field images of day-8 hatched blastocysts generated by somatic cell nuclear transfer using *IFNT*-KO and WT donor cells. **b,** Representative images of *IFNT*-KO and WT embryos after extended in vitro culture on days 15 and 19. For extended culture, individual blastocysts were cultured separately in individual medium drops. Note the limited elongation and onset of degenerative changes typically observed in bovine embryos during extended culture under standard in vitro conditions.

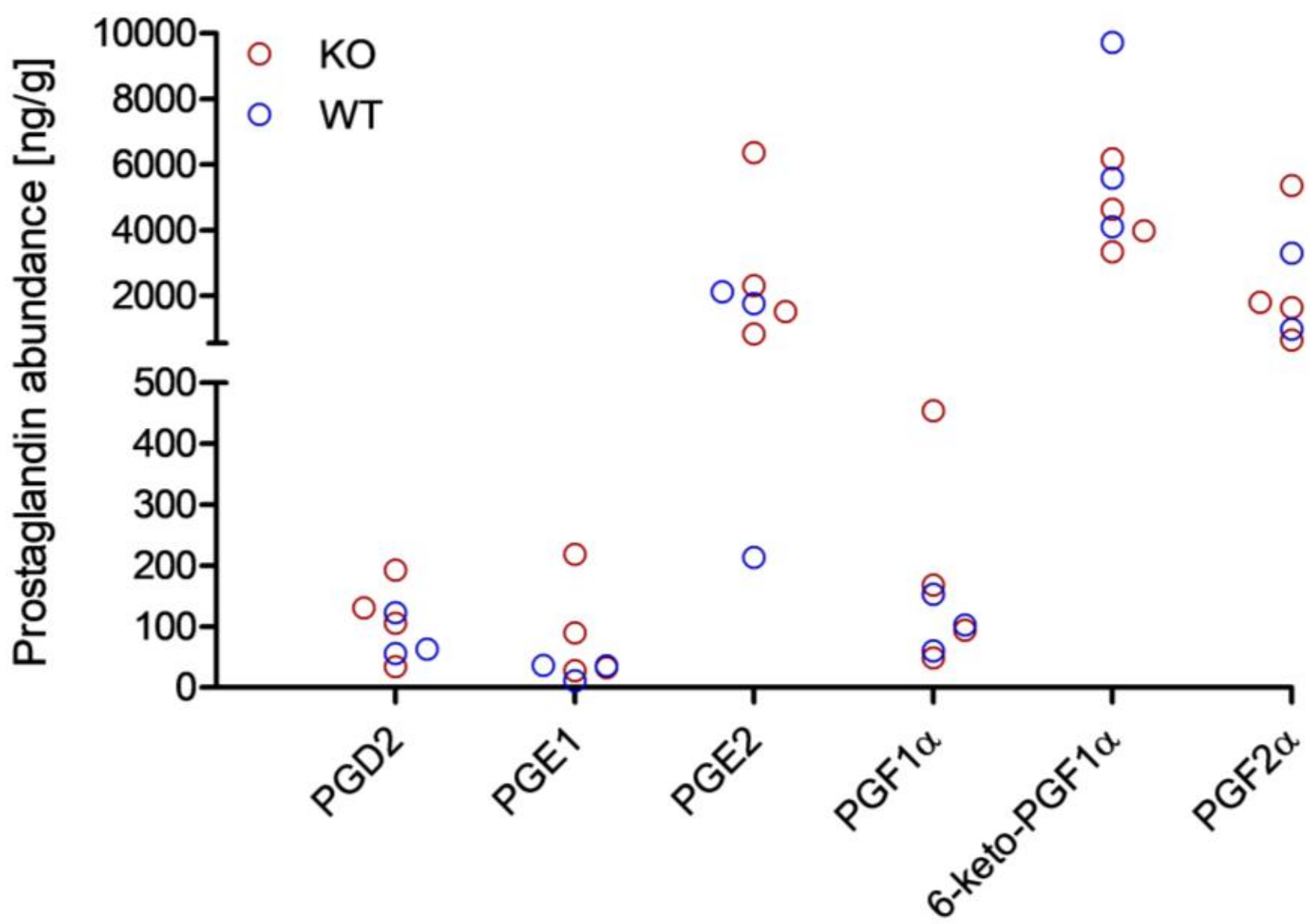

Supplementary Figure 2 | Quantification of key prostaglandins in day-18 *IFNT*-KO and WT conceptus tissues by LC/ESI-MS/MS. Quantification of PGD_2, PGE_1, PGE_2, $PGF_1\alpha$, PGF1α-6-keto, and $PGF_2\alpha$ in day-18 conceptus tissues from *IFNT*-KO (n = 4 biological replicates) and WT (n = 3 biological replicates) groups. Data points represent individual biological replicates. Differences between groups were evaluated using a two-sided Mann–Whitney *U* test. No statistically significant differences were observed between genotypes for any of the analyzed prostaglandins ($p > 0.05$). Statistical analysis and graph generation were performed using GraphPad Prism (v10.1.6). Source data are provided at the end of the Supplementary Information.

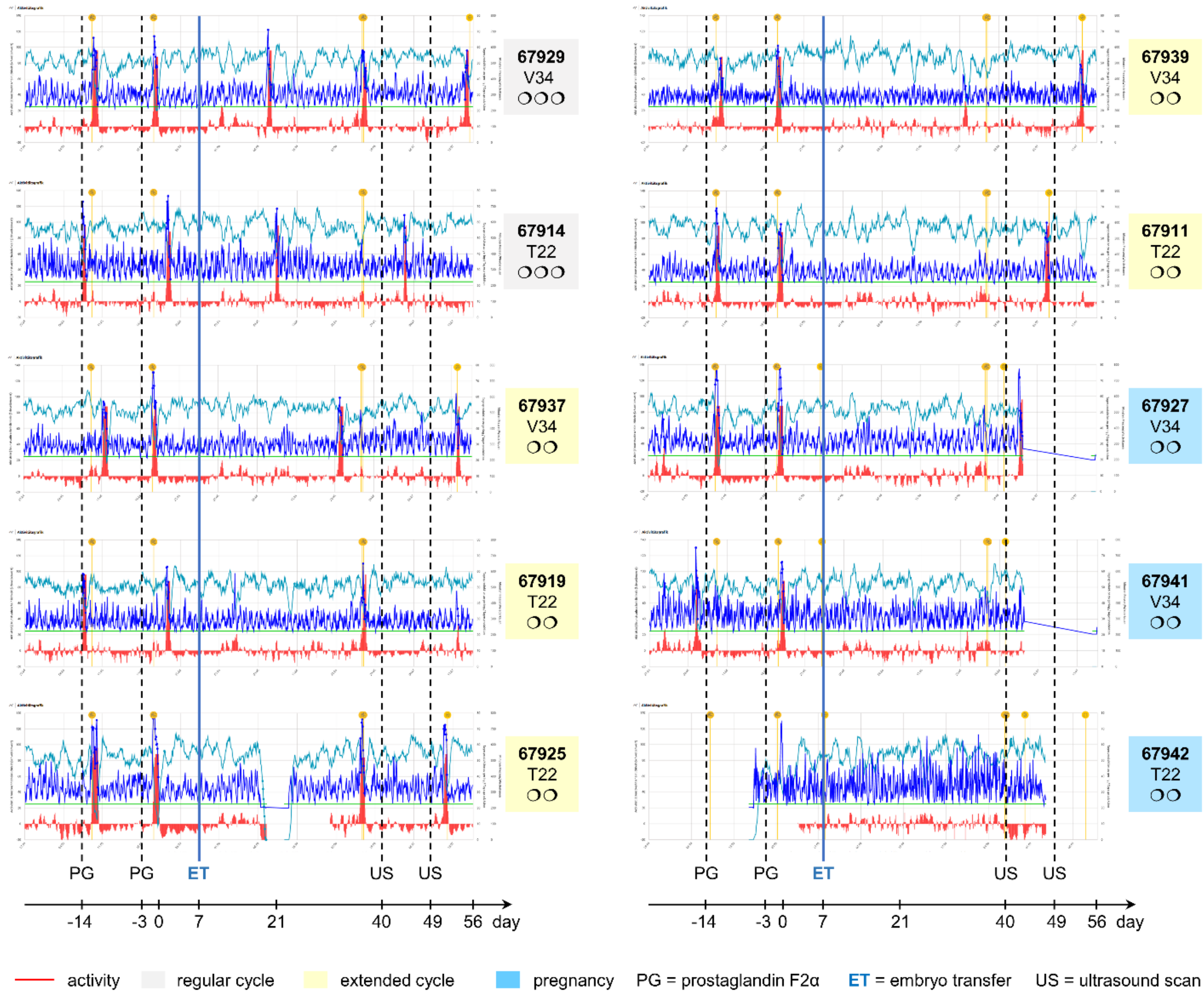

Supplementary Figure 3 | Automated estrous cycle profiles and pregnancy outcomes following transfer of *IFNT*-KO embryos. Recipient heifers were synchronized with two injections of prostaglandin $F_2\alpha$ (PG) on days −14 and −3, with day 0 defined as the day of standing estrus. On day 7 of the synchronized cycle, 10 recipients received SCNT-derived *IFNT*-KO blastocysts (eight recipients received two embryos each and two recipients received three embryos each). Recipient activity was continuously monitored using an automated movement-recording system, and time-series activity profiles were generated to detect estrous behavior. Two recipients (67914 and 67929), each of which received three embryos, returned to estrus at the expected interval (days 20–21). In contrast, the remaining eight recipients exhibited prolonged luteal phases, consistent with the establishment of pregnancy. Transrectal ultrasonography on days 40 and 49 confirmed twin pregnancies in three recipients (67927, 67941, and 67942).

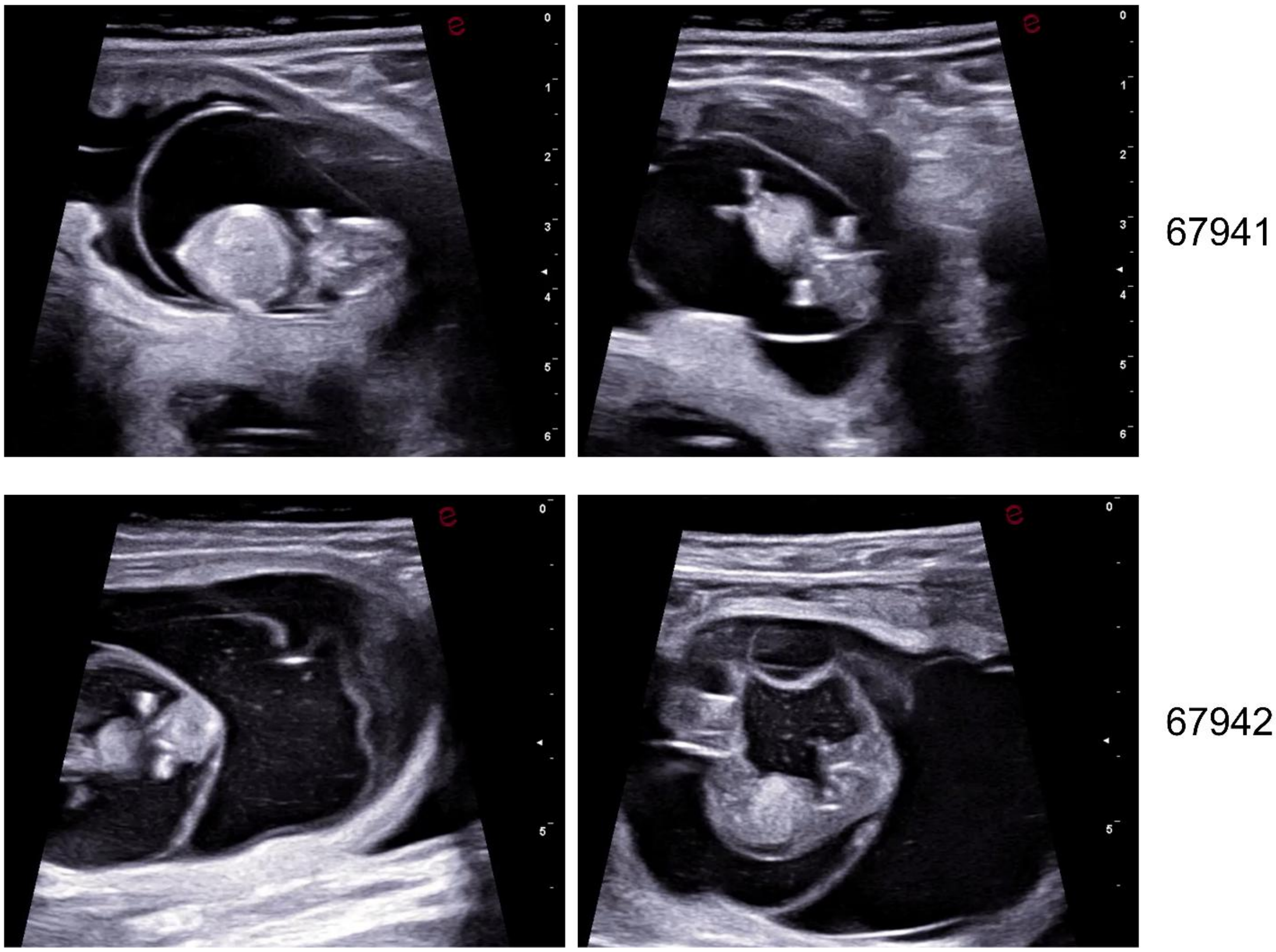

Supplementary Figure 4 | Ultrasonographic confirmation of twin pregnancies following transfer of *IFNT*-KO blastocysts. Transrectal ultrasonography on gestational day 49 confirmed twin pregnancies in three recipients. Representative images from recipients 67941 and 67942 show two viable fetuses in each uterus. Fetal heartbeats are shown in **Supplementary Video 1**.

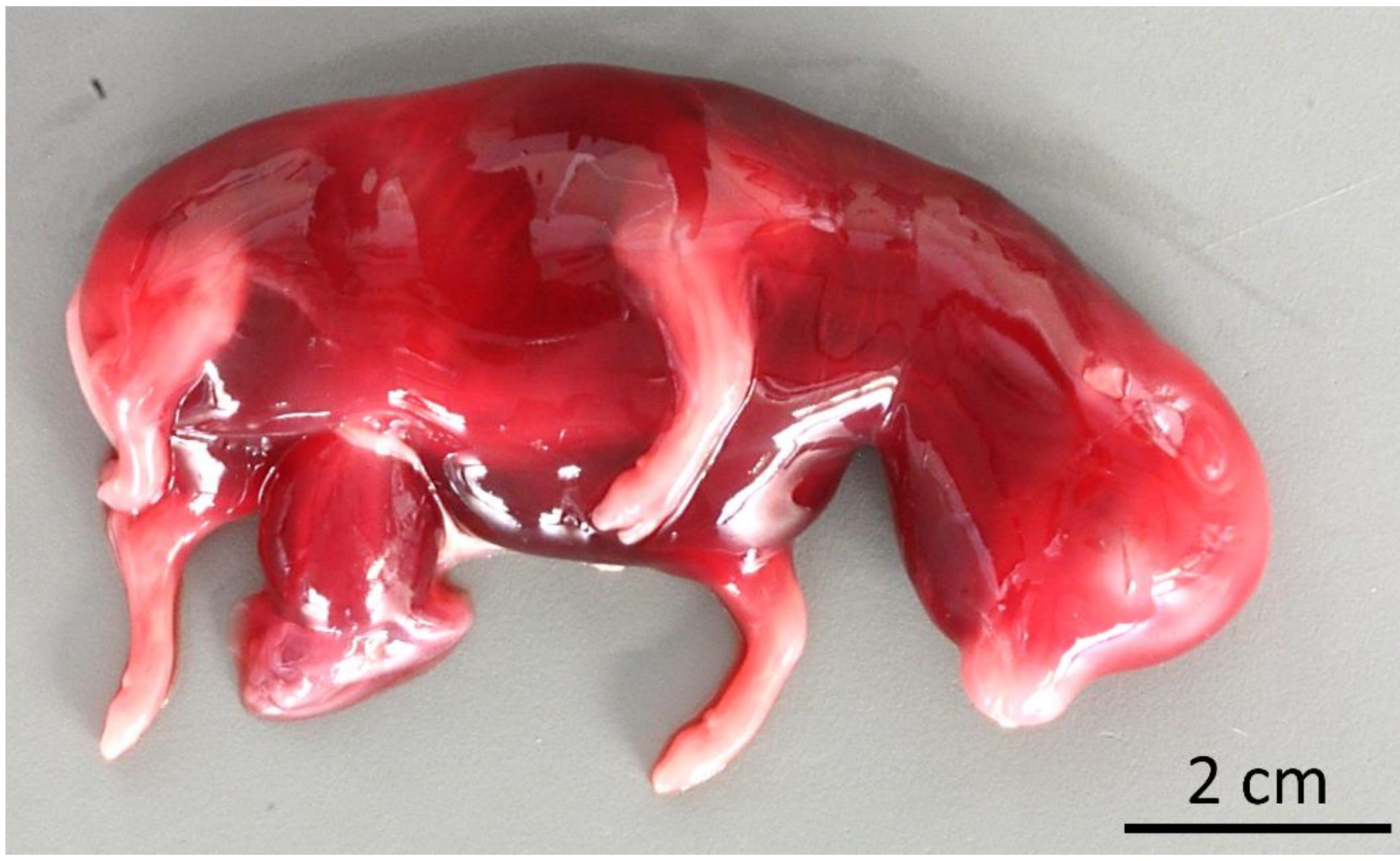

Supplementary Figure 5 | *IFNT*-KO fetus recovered on gestational day 75, exhibiting cloning-associated abnormalities. The fetus, generated by SCNT and carrying a complete *IFNT* knockout, was a clonal sibling of the fetus shown in **Fig. 4g** and was recovered from recipient 67941. Although the fetus was of an overall size appropriate for its gestational age, it exhibited generalized hydrops fetalis, a developmental abnormality frequently observed following SCNT.

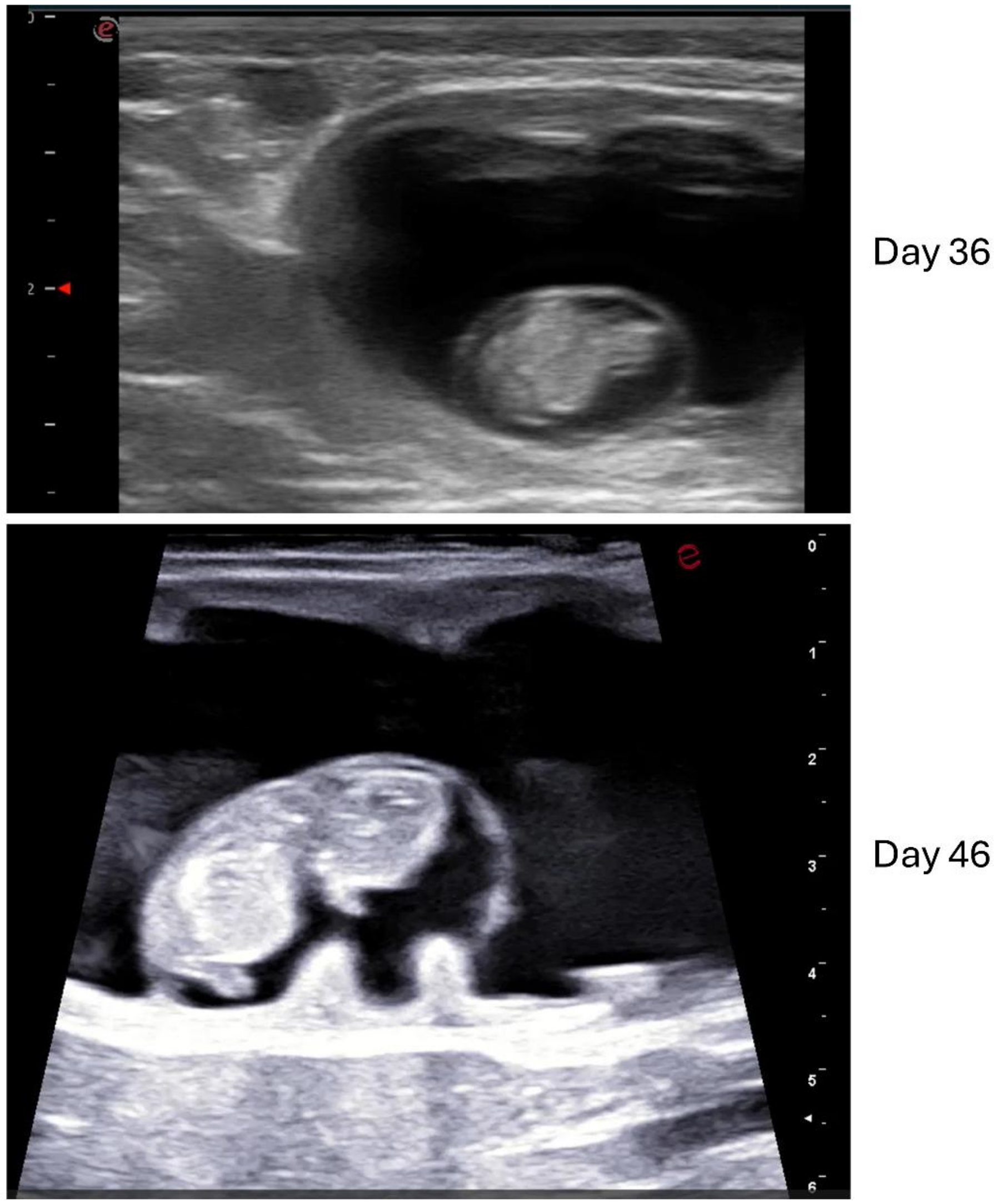

Supplementary Figure 6 | Ultrasonographic confirmation of pregnancy following transfer of a single *IFNT*-KO blastocyst. Transrectal ultrasonography on gestational days 36 and 46 confirmed a viable singleton pregnancy in recipient 19171. Representative images of a viable fetus with a detectable heartbeat, as shown in **Supplementary Video 2**.

Supplementary Table 1 | In vitro development of somatic cell nuclear transfer (SCNT) embryos produced from *IFNT*-KO and WT cells.

Donor cells	Exp.	Date	Oocytes used	Fused SCNT complexes		Cleaved SCNT embryos		Day 7 blastocysts	
			n	n	(%)[a]	n	(%)[b]	n	(%)[c]
KO	1	21.05.2025	108	104	96	91	88	48	53
KO	2	10.07.2025	32	30	94	15	50	8	53
KO	3	24.07.2025	28	24	86	13	54	5	39
KO	4	12.08.2025	28	25	89	18	72	5	28
KO	5	25.09.2025	16	12	75	12	100	10	83
		**Total**	**212**						
WT	1	10.07.2025	34	32	94	13	41	6	46
WT	2	24.07.2025	26	23	89	16	70	3	19
WT	3	12.08.2025	24	21	88	10	48	4	40
WT	4	02.10.2025	47	43	92	30	70	9	30
WT	5	06.11.2025	13	11	85	6	55	4	67
		**Total**	**144**						

In total, five independent SCNT sessions were performed using *IFNT*-KO donor cells and five sessions using WT donor cells. Detailed embryo numbers, fusion rates, cleavage rates, and blastocyst formation rates for every session are provided; [a]based on the number of oocytes used for SCNT; [b]based on the number of fused SCNT complexes; [c]based on the number of cleaved SCNT embryos.

Supplementary Table 2 | Pregnancy outcomes following transfer of *IFNT*-KO blastocysts (multiple embryo transfers).

Recipient ID	Number of blastocysts transferred	Cycle length (days)[a]	Pregnancy check by ultrasound[b]
67929	3	20	Not pregnant
67914	3	21	Not pregnant
67937	2	33	Not pregnant
67919	2	37	Not pregnant
67925	2	37	Not pregnant
67939	2	54	Not pregnant
67911	2	48	Not pregnant
67927	2	No estrus observed	Twin pregnancy (Term)[c]
67941	2	No estrus observed	Twin pregnancy (Day 75)
67942	2	No estrus observed	Twin pregnancy (Day 50)

Ten synchronized recipient heifers received either two or three *IFNT*-KO blastocysts. Cycle length was monitored using automated accelerometer-based activity systems (day of estrus before embryo transfer = day 0). Pregnancy status was confirmed by transrectal ultrasonography starting on day 40 post-estrus. [a]Cycle length was measured based on accelerometer activity, as shown in Supplementary Fig. 3; [b]the first ultrasound examination was performed on day 40 post-estrus; [c]two healthy calves were born on February 26, 2026.

Supplementary Table 3 | Clinical examination of *IFNT*-null calves on day 15 post-partum.

**Parameter**<br>**(normal reference value)**	**Calf # 38345**	**Calf # 38346**
Rectal temperature (38.5 - 39.5 °C)	39.2 °C	39.0 °C
Heart rate (165.64 ± 17.61 bpm)<br>https://doi.org/10.5513/JCEA01/26.2.4520	180 beats/min (strong, no murmurs)	160 beats/min (strong, no murmurs)
Umbilicus	Normal, umbilical cord dry	Normal, umbilical cord fallen off
Feces	Ocher-colored, thick pasty	Ocher-colored, medium pasty
PA / SA<br>(patent ductus arteriosus/septum)	Negative bilaterally	Negative bilaterally
Abdominal wall	Soft	Soft
Skin turgor	Normal	Normal
Mucous membranes	Pale pink, no erosions	Pale pink, no erosions
Joints	Normal	Normal
Ears	Normal	Normal
Hair coat	Fine, well-formed	Fine, well-formed
General condition	Bright, playful	Bright, alert

Supplementary Table 4 | Pregnancy outcomes following single *IFNT*-KO blastocyst transfer.

Recipient ID	Cycle length (days)[a]	Pregnancy check by ultrasound[b]
19158	21	Not pregnant
19170	No follow-up<br>(severe knee injury and medication)	Not pregnant
19175	21	Not pregnant
19171	No estrus observed	Pregnancy confirmed

Four synchronized recipients received a single *IFNT*-KO blastocyst. [a] First ultrasound was performed on day 36, with a follow-up on day 46.

Source Data for Supplementary Figure 2 | Key prostaglandins in day-18 conceptus tissues (ng/g)

	IFNT-KO (*n* = 4)				WT (*n* = 3)		
**Prostaglandin**	1	2	3	4	1	2	3
PGD2	130.61	105.62	34.03	192.97	123.07	55.8	63.26
PGE1	89.68	32.54	27.98	219	35.58	36.05	11.13
PGE2	2309.69	1517.58	831.98	6363.15	2127.81	1756.61	213.54
PGF1α	168.48	93.75	48.93	454.21	152.75	60.36	102.89
PGF1α-6-keto	3336.3	6176.38	4639.57	3983.39	4098.96	5586.29	9727.05
PGF2α	1796.85	1637.25	650.49	5363.14	3303.05	980.98	1074.87
