# Supplementary material for: Pregnancy establishment in cattle without embryonic interferon tau
Source: Nat Commun. 2026 Oct 8;17:10609. doi: 10.1038/s41467-026-78446-4 (PMC13645731; doi:10.1038/s41467-026-78446-4)
Supplement: Supplementary file 4 [file 41467_2026_78446_MOESM2_ESM.pdf]

Description of Additional Supplementary Files

File name: Supplementary Data 1
Description: 1a | Normalized label-free quantification (LFQ) intensities of IFNT peptides in conditioned media. **1b |** Sum of LFQ intensities AA (log2).

File name: Supplementary Data 2
Description: 2a | Log2 label-free quantification (LFQ) intensities of all peptides detected in IFNT-KO and wild-type (WT) conceptuses. IFNT peptides were exclusively identified in WT samples and were completely absent in all IFNT-KO conceptuses. **2b |** Results of volcano plot analysis of IFNT-KO vs. wild-type (WT) conceptuses.

File name: Supplementary Data 3
Description: Normalized label-free quantification (LFQ) intensities for each IFNT-specific peptide in IFNT-KO and wild-type (WT) conceptuses. IFNT peptides were exclusively identified in WT samples and were completely absent in all IFNT-KO conceptuses.

File name: Supplementary Data 4
Description: 4a | RNA-seq read counts for IFNT-KO and WT conceptuses. **4b |** Statistics for differential gene expression between IFNT KO and WT concepti as obtained with the R package edgeR (v4.8.2).

File name: Supplementary Data 5
Description: Log2 label-free quantification (LFQ) intensities for the prostaglandin E synthases PTGES, PTGES2, and PTGES3 proteins (top) and normalized mRNA counts (bottom) in *IFNT*-KO and wild-type (WT) conceptuses. No significant differences were observed between groups for any isoform (two-tailed Student's t-test).

File name: Supplementary Data 6
Description: 6a | Log2 label-free quantification (LFQ) intensities of all peptides detected in endometrium samples exposed to *IFNT*-KO vs. WT conceptuses. **6b |** Results of volcano plot analysis of endometrium samples exposed to *IFNT*-KO vs. WT conceptuses.

File name: Supplementary Data 7
Description: Results of STRING functional enrichment analysis using the Reactome database.

File name: Supplementary Video 1
Description: Transrectal ultrasonography of recipients following transfer of two *IFNT*-KO embryos. Transrectal ultrasonography on day 40 of gestation confirmed twin pregnancies in three recipient heifers (67927, 67941, 67942) following transfer of two *IFNT*-KO blastocysts.

File name: Supplementary Video 2
Description: Transrectal ultrasonography of a recipient following transfer of a single *IFNT*-KO embryo. Transrectal ultrasonography on day 46 of gestation confirmed an ongoing pregnancy in a recipient heifer following transfer of a single *IFNT*-KO blastocyst.
